# Depression screening and mental health outcomes in children and adolescents: a systematic review protocol

**DOI:** 10.1186/2046-4053-1-58

**Published:** 2012-11-24

**Authors:** Brett D Thombs, Michelle Roseman, Lorie A Kloda

**Affiliations:** 1 Jewish General Hospital, Institute for Community and Family Psychiatry, 4333 Cote Ste Catherine Road, Montréal, QC, H3T 1E4, Canada; 2 Department of Epidemiology, Biostatistics and Occupational Health, McGill University, Montréal, QC, Canada; 3 Department of Medicine, McGill University, Montréal, QC, Canada; 4 Department of Educational and Counselling Psychology, McGill University, Montréal, QC, Canada; 5 School of Nursing, McGill University, Montréal, QC, Canada; 6 Lady Davis Institute for Medical Research, Jewish General Hospital, Montréal, QC, Canada; 7 Library, McGill University, Montréal, QC, Canada

**Keywords:** Adolescents, Children, Depression, Screening

## Abstract

**Background:**

Depression is an important cause of disability among children and adolescents. Depression screening is one possible method for managing depression, and screening programs have been initiated in some school and medical settings. However, in 2005, the Canadian Task Force on Preventive Health Care and the United Kingdom National Institute of Clinical Excellence did not recommend depression screening among children and adolescents. By contrast, in 2009, the United States Preventive Services Task Force recommended that all adolescents, but not younger children, be screened for depression in medical settings with integrated depression management services, although no trials of screening were identified. The objectives of this systematic review are to evaluate in children and adolescents the accuracy of depression screening tools; depression treatment efficacy; whether depression screening improves depression outcomes; and potential harms related to depression interventions and screening.

**Methods/design:**

Data sources will include the bibliographic databases MEDLINE, Cochrane CENTRAL, PsycINFO, EMBASE, LILACS and Web of Science, supplemented by reference harvesting of eligible articles, relevant systematic reviews, relevant guidelines and recommendations, and selected journals, and by searches for unpublished studies. Eligible studies will report data for children and adolescents aged 6 to 18 years. Eligible diagnostic accuracy studies must compare a depression screening tool to a validated diagnostic interview for major depressive disorder and report diagnostic accuracy data. Eligible treatment studies must be randomized controlled trials of pharmacological, psychotherapeutic, or other depression treatments commonly available for children and adolescents in pediatric, primary-care, and family medicine settings. Eligible screening studies must be randomized controlled trials that compare depression outcomes between children or adolescents who underwent depression screening versus those who did not. Studies of harms will include randomized controlled trials and observational studies that evaluate harms from depression screening or treatment. Two investigators will independently review titles and abstracts, followed by full article review. Disagreements will be resolved by consensus. Two investigators will independently extract the data, with discrepancies resolved via consensus.

**Discussion:**

The proposed systematic review will determine whether there is sufficient evidence of benefits in excess of harms and costs to support screening for depression in childhood and adolescence.

## Background

Depression in children and adolescents is a disabling condition that is associated with long-term mental and physical health problems [1]. Screening for depression is one possible solution to improve depression management. In 2005, however, the Canadian Task Force on Preventive Health Care (CTFPHC) determined that there was not sufficient evidence on health outcomes from depression screening to recommend the practice among children and adolescents in primary health care settings [2]. Consistent with this, a 2005 guideline from the United Kingdom’s National Institute for Health and Clinical Excellence (NICE) concluded that universal screening was not advised based on available evidence [3]. In 2009, by contrast, the United States Preventive Services Task Force (USPSTF) concluded that adolescents, but not younger children, should be routinely screened for depression in primary health care settings ‘when systems are in place to ensure accurate diagnosis, psychotherapy (cognitive-behavioral or interpersonal), and follow-up’ (page 1223) [4]. This recommendation was made based on a systematic review of the literature through May 2006 even though, as the USPSTF recognized, there was little data on the accuracy of screening tools and no data to compare health outcomes between screened and unscreened adolescents [1]. In the more than 5 years since that review, a substantial amount of research relevant to depression screening in children and adolescence has been published.

Screening is potentially costly in health care resources and has the potential to cause harm to some children and adolescents who may be diagnosed or treated inappropriately. An important empirical question is whether screening, given these concerns, would be more effective than alternative care models that do not rely on universal screening. As described in a recent critique of calls for depression screening, there are a number of reasons why simply assuming that depression screening would result in more benefit than harm may not be reasonable, including high false positive rates if screening tools are not sufficiently precise, generally small depression treatment effects, and the inconsistent quality of routine care that is often provided [5]. Thus, the objective of the proposed systematic review is to evaluate the mental health effects of screening children and adolescents aged 6 to 18 years for major depressive disorder (MDD) in medical or community settings. To do this, we identified the following key questions based on well-established criteria delineated by the World Health Organization [6] and the United Kingdom National Screening Committee [7], as well as the methodological framework of the USPSTF for evaluating screening programs [8]:

***Key Question*#*1*:** What is the accuracy of depression screening instruments to detect cases of MDD among (a) children and (b) adolescents?

***Key Question*#*2*:** Is treatment of MDD during (a) childhood and (b) adolescence effective in improving symptoms of depression?

***Key Question*#*3*:** Is depression screening during (a) childhood and (b) adolescence more effective than usual care in (i) improving depressive symptoms or (ii) reducing the number of MDD diagnoses?

***Key Question*#*4*:** What are the potential harms associated with depression screening, including treatment, during (a) childhood and (b) adolescence?

### Major depressive disorder in children and adolescents

Depression is primarily characterized by the core symptoms of persistent sad mood or irritability and/or a loss of interest or pleasure in activities that are normally enjoyed. Among children and adolescents, irritability may be more prominent than sad mood, and tantrums and other disruptive behavior may also be important markers. In North America, MDD is diagnosed among children and adolescents based on criteria established by the American Psychiatric Association and delineated in the 4th Edition of the Diagnostic and Statistical Manual (DSM-IV) [9]. Criteria for a diagnosis of MDD include having at least five of nine depressive symptoms for 2 weeks or more, at least one of which must be depressed mood, including irritability, or loss of interest in activities. Symptoms must cause significant distress or impairment in daily function. MDD should be distinguished from bipolar disorder, which is characterized by abnormal and disruptive elevated moods, in addition to depression. The DSM-IV also includes dysthymic disorder, which is a longer lasting, but less severe, mood manifestation than MDD, as well as minor depressive disorder, which requires only two, rather than five, depressive symptoms. This review, consistent with earlier reviews by the CTFPHC [2] and the USPSTF [1] will assess evidence relevant to screening for MDD, but not dysthymic disorder or minor depression, for which treatment options and efficacy are much less well delineated. It will also include studies relevant to screening for depression based on the International Classification of Diseases (ICD) definition of depression, which is similar to the DSM-IV definition [10].

### Prevalence and burden of depression in children and adolescents

A 2006 meta-analysis that synthesized data from over 60,000 adolescents aged 13 to 18 years estimated the point prevalence of MDD to be 5.6% in the community with rates slightly higher among girls than boys [11]. In Canada, a study of almost 18,000 respondents to the National Population Health Survey (1995 to 1996) found that 4.8% of boys and 8.7% of girls had an episode of MDD in the past year. Among youth aged 12 to 14 years, the rates were 2.7% for boys and 2.6% for girls. For adolescents aged 15 to 19 years, 6.1% of boys and 12.5% of girls had experienced at least one episode [12]. Compared to community samples, in medical settings the rate may be up to twice as high [13]. Some research has suggested that the lifetime prevalence of an episode of MDD among adolescents may be as high as 20% [1,14,15]. In Canada, however, a study that used data from the Canadian Community Health Survey Cycle 1.2 reported a lifetime prevalence rate among almost 3,000 adolescents aged 15 to 19 years of 7.6% for MDD, including 4.3% for males and 11.1% for females [16]. Among youth under age 13, the point prevalence of MDD has been estimated to be approximately 3% [11].

MDD in childhood and adolescence is associated with many negative outcomes, including behavioral problems and poor school performance, early pregnancy, and impaired social, work, and family functioning in adolescence and into adulthood [1]. Many studies have shown that patients with depression in adulthood had at least one episode of MDD in childhood or adolescence, and there is a high rate of recurrence among youth with MDD [1,15,17-19]. Despite this substantial burden, relatively few children and adolescents with depression receive treatment [20-22].

### What is depression screening and when should it be recommended?

Screening is a preventive strategy that is traditionally used to detect disease in patients who otherwise have no signs or symptoms. Screening programs are premised on the assumption that early detection of disease will enable earlier and more effective intervention. Unlike screening for most other medical diseases, however, screening for depression does not seek to achieve early identification of pre-symptomatic cases that will subsequently evolve into psychiatric disorder. Rather, screening for depression involves the use of depression symptom questionnaires or small sets of questions about depression to identify patients who may have current depression, but who have not sought treatment and whose depression has not already been recognized by health care providers. Patients identified as possible cases need to be further assessed and, if appropriate, offered treatment. Depression screening is potentially useful only to the extent that it improves patient outcomes beyond any treatment that is provided as part of standard care. Thus, to be successful, a screening program must identify a significant number of depressed patients who are not already diagnosed with depression, engage those patients in treatment, and obtain sufficiently positive treatment results to justify costs and potential harms from screening [5].

In 1968, the World Health Organization issued a report that delineated criteria to determine whether conditions are suitable for screening [6], and the main elements of those criteria continue to be used today [23]. Generally, it is reasonable to consider screening when the condition in question is important and prevalent, can be effectively treated, and cannot be readily detected without screening. Further, screening methods should be accurate and carry only a tolerably small risk of false positive results, which could lead to unnecessary diagnostic testing, adverse effects, costs of inappropriate treatment, and to sequelae of being incorrectly labeled, such as stigma. False reassurance for false negatives may also need to be considered in some circumstances. The principal criterion is that there must be evidence that benefits from screening outweigh potential harms. Ideally, benefits in excess of potential harms should be demonstrated consistently in well-conducted randomized controlled trials (RCTs) with sufficiently long follow-up to cover the time horizon of important patient-oriented outcomes.

### Current practices in depression screening in childhood and adolescence

Studies from community [24] and health maintenance organization [25] settings in the United States published in 2000 and 2001 suggested that 40% to 60% of adolescent patients may be screened for depression based on physician report, but this number fell to only 3% when medical charts were examined for documentation of screening [24]. More recently, TeenScreen, an American organization based at Columbia University, has aggressively promoted widespread mental health screening for adolescents and has reported that they received more than 400,000 requests for screening questionnaires from schools, primary-care physicians and managed-care organizations in 2010 alone [26]. There are numerous reports of mental health screening supported by TeenScreen being conducted routinely in medical practices and across entire school districts in the United States [27-29]. In Canada, the governments of Alberta and Manitoba, for instance, have called for widespread depression screening in school settings as part of long-term plans to improve youth mental health [30,31].

### Existing guidelines and recommendations

In Canada, the last guideline statement on the topic was a CTFPHC recommendation from 2005 [2]. The CTFPHC recommendation focused on adult depression, but noted that, consistent with previous findings from the USPSTF, there was not enough evidence to support a recommendation for screening in child or adolescent medical care settings. Similarly, a 2005 NICE guideline on depression management for children and youth in the United Kingdom emphasized the importance of better and more consistent care, but concluded that the evidence needed to support a screening recommendation was not available [3]. More recently, in 2009, the USPSTF issued a recommendation that adolescents in medical settings should be screened for depression in primary-care settings when integrated care systems are in place to ensure accurate diagnosis, competent psychotherapeutic and medical support, and follow-up [4]. The recommendation document focused on the prevalence and burden of depression among adolescents, as well as the existence of screening tools and treatments, but not on evidence from any RCT that mental health outcomes improved among youth who were screened compared with youth who were not screened for depression [4]. Calls for depression screening have gone beyond medical settings. The 2003 United States President’s New Freedom Commission on Mental Health called for screening in primary medical care, school, and child welfare settings as the key to reducing the community burden of depression [32], and school-based screening programs have been implemented in Canada [30]. Existing guidelines and recommendations, however, have not directly addressed whether school-based screening outside of the context of health care settings is recommended.

### Previous systematic reviews and meta-analyses

A number of reviews have assessed the accuracy of screening tools for detecting MDD, the efficacy of treatments, and harms that may be associated with depression treatment in children or adolescents (see Additional file 1). Only one review [1], a United States Agency for Healthcare Research and Quality (AHRQ) report, which was done in conjunction with a USPSTF guideline [4], has assessed the various components of a depression screening program for children or adolescents (accurate screening tools, effects of treatment, effects of screening, potential harms) and whether there was evidence that mental health outcomes were better for screened versus non-screened children and adolescents. The review was published in 2009, but only included studies through a search that was done through May 2006. The review included nine studies on the accuracy of six different depression screening tools, but reported that all had serious methodological flaws, including non-random patient selection, excessive delays between administration of screening tools and diagnostic interviews, high levels of attrition, poor reporting of methods and results, and small samples. No studies were rated as being of good quality. The review identified 18 fair- to good-quality RCTs on treatment for MDD with psychotherapy, medication, or a combination of psychotherapy and medication, and concluded that treatments were generally effective, but that not all medications appeared to work well. There were no studies identified that examined mental health outcomes among children or adolescents who were screened compared to children and adolescents who were not screened.

## Methods/design

This systematic review has been funded by the Canadian Institutes for Health Research (Funding Reference Number KA1 – 119795). The protocol has been registered in the PROSPERO prospective register of systematic reviews (CRD42012003194).

The review methodology was designed to be consistent with reporting guidelines described in the Meta-analysis of Observational Studies in Epidemiology statement [33] and the Preferred Reporting Items for Systematic Reviews and Meta-analyses statement [34,35]. Key questions for this systematic review are based on the USPSTF analytic framework [8,36]. The USPSTF logic model describes a process for determining whether screening benefits likely outweigh harms, including the specific populations, screening procedure, and health outcomes to be considered [8]. The model assumes that patients with undetected depression undergo screening, which classifies patients as cases or non-cases of MDD, and that the evaluation of the accuracy of screening tests is a key component. Treatment studies are evaluated to determine the degree to which they improve symptoms of depression. Trials of actual screening programs, in which health outcomes are evaluated for screened versus unscreened children and adolescents are, if they exist, the final standard by which the value of screening can be tested in terms of its ability to improve depression outcomes and produce benefits to children and adolescents that exceed potential harms from screening.

### Search strategy

Four distinct sets of searches will be conducted, one for Key Question #1 (accuracy of screening tools), one for Key Question #2 (effects of treatment), one for Key Question #3 (effects of screening), and one for Key Question #4 (harms). To identify studies relevant to Key Question #1 (accuracy of screening tools), the MEDLINE, EMBASE, PsycINFO, HAPI, and LILACS databases will be searched. For Key Questions #2 (effects of treatment), #3 (effects of screening), and #4 (harms of screening), the MEDLINE, EMBASE, PsycINFO, Cochrane CENTRAL, and LILACS databases will be searched. All search strategies have been peer-reviewed. There will be no language restrictions. See Additional file 2 for specific search terms that will be used.

Searches will be run from January 2006 to the present because an AHRQ systematic review on depression screening in children and adolescents [1] searched to May 2006. Eligible studies from that review will be included in the present review. For the effects of depression treatment, the previous AHRQ review [1] included only studies on the effects of selective serotonin reuptake inhibitors (SSRIs) and psychotherapy. However, serotonin-norepinephrine reuptake inhibitors (SNRIs), other non-SSRIs (bupropion, mirtazapine, trazodone), and exercise are also treatments for depression that may be commonly used with children or adolescents in general pediatric settings or primary-care and family medicine settings. Thus, for Key Question #2 (effects of treatment), an additional search with no date restriction will be run for studies on the effects of SNRIs, a select group of non-SSRIs (bupropion, mirtazapine, trazodone), and exercise.

In addition, manual searching will be done for the period of 3 months prior to the final database search on the references of eligible original articles, relevant systematic reviews and meta-analyses, and selected journals, including journals in psychiatry, psychology, and pediatrics. To identify unpublished or ongoing trials, we will use methods that include reviewing trial registries and results databases (for example, ClinicalTrials.gov), pharmaceutical industry trial registries and results databases, regulatory agency online databases, regulatory agency submissions, litigation documents, and conferences abstracts, as well as contacting trialists and sponsors for unpublished or ongoing trials [37].

### Identification of eligible studies

Eligible studies will include children and adolescents aged 6 to 18 years. We will include studies conducted in general medicine clinics, schools, and community settings. Studies including medically ill children are eligible. Studies of college and university populations will be excluded, as college and university students have a different pathway to mental health treatment than adolescents under their parents' care.

***Key Question*#*1*(*accuracy of screening tools*):** Studies on the accuracy of screening tools will be included if they compared a screening instrument with a valid criterion standard, defined as a DSM diagnosis of MDD or an ICD diagnosis of depressive episode based on a validated diagnostic interview procedure, and if they reported data allowing determination of sensitivity and specificity, positive predictive value, and negative predictive value. Examples of validated psychiatric interviews that have been used in assessments of children or adolescents include, but are not limited to, the Composite International Diagnostic Interview, the Diagnostic Interview for Children and Adolescents, the Revised Diagnostic Interview Schedule for Children, and the Schedule for Affective Disorders and Schizophrenia for School-Aged Children. Studies that assess broader diagnostic categories, such as any depressive disorder or dysthymia, will be included only if they report data for MDD separately. Studies must administer the screening tool and the diagnostic assessment within 2 weeks of each other to be eligible. Studies in which only parent- or teacher-completed measures, but no child or adolescent self-report measures, are compared to a diagnosis of MDD will be excluded. Studies will be excluded if a cutoff score above a threshold on a depression screening tool is used as an eligibility criterion to receive assessment for MDD with a validated structured interview. Studies conducted in high-risk populations where many or most children and adolescents may have a psychiatric disorder, such as in psychiatric or youth-protection settings, will be excluded.

***Key Question*#*2*(*effects of treatment*):** Studies on treatment will include RCTs with placebo or usual care controls that evaluated pharmacological, psychotherapeutic, or other interventions that would typically be available to children or adolescents in general pediatric settings or primary-care and family medicine settings as treatment for depression as diagnosed with a validated psychiatric interview and DSM criteria for MDD or ICD criteria for a depressive episode. Specifically, eligible interventions will include SSRIs (fluoxetine, sertraline, paroxetine, citalopram, escitalopram, and fluvoxamine), SNRIs (venlafaxine, duloxetine, desvenlafaxine), other non-SSRIs prescribed for children or adolescents with depression (bupropion, mirtazapine, trazodone), psychotherapy, educational interventions, and exercise. We will require, as an eligibility criterion, a DSM diagnosis of MDD or ICD diagnosis of depressive episode based on a valid diagnostic interview because unassisted clinician diagnoses have poor reliability and because a large proportion of patients scoring above cutoffs on self-report questionnaires do not have MDD. Head-to-head trials of different interventions without a comparison to usual care or placebo are not eligible.

***Key Question*#*3*(*effects of screening*):** Eligible studies will be RCTs that compared depression outcomes between children or adolescents who underwent depression screening and those who did not. Studies in which comprehensive depression care programs were provided to children or adolescents with depression as part of the screening program will be included if children and adolescents in the unscreened group could also access these treatment programs if identified as depressed by means other than screening. Otherwise, it will not be possible to disaggregate effects of screening from effects of providing comprehensive depression management. Changes in rates of depression recognition and treatment will be noted, but not included as depression outcomes. This is because increased treatment is not a benefit and, if improved depression outcomes are not obtained, exposes children and adolescents to costs and potential harms of treatment without benefit. Screening is defined per the United Kingdom National Screening Committee’s definition. Thus, eligible screening trials had to include a case identification strategy based on an *a priori* defined cutoff score on a depression screening tool to make decisions regarding further assessment or treatment.

***Key Question*#*4*(*harms*):** Studies that document harms from depression treatment or screening will include RCTs, as well as observational studies.

Two investigators will independently review titles and abstracts for eligibility with full-text review of articles identified as potentially eligible by one or both. Disagreements after full-text review will be resolved by consensus. Chance-corrected agreement will be assessed with Cohen’s κ. See Additional file 3 for the coding manual.

### Data extraction and outcomes

Two reviewers will independently extract relevant data from each eligible article and enter the data directly into formatted Excel spreadsheets. Entries will be compared for accuracy and any discrepancies will be resolved by consensus. Authors of original studies will be contacted if necessary to clarify inconsistencies in reported results. See Additional file 4 for variables included in data extraction templates.

***Key Question*#*1*(*accuracy of screening tools*):** Outcomes reported will include sensitivity, specificity, positive predictive value, and negative predictive value, based on child or adolescent self-report measures.

***Key Questions*#*2*(*effects of treatment*)*and*#*3*(*effects of screening*):** The primary outcome will be standardized mean difference effect size based on a continuous measure of depressive symptoms. When multiple depression outcomes are reported, primary outcomes as identified in each study will be given highest priority. Then observer-rated scales will be prioritized over self-report measures. Outcomes will be reported as intent-to-treat analyses where possible and will be prioritized over those presented for completers only.

***Key Question*#*4*(*harms*):** Outcome reporting will be based on harms identified from the review, but we expect the main potential harm to be suicidal ideation.

### Appraisal of risk of bias

For Key Question #1 (accuracy of screening tools), we will use the Quality Assessment of Diagnostic Accuracy Studies-2 tool [38]. This tool incorporates assessments of risk of bias across four core domains: patient selection, the index test, the reference standard, and the flow and timing of assessments. For Key Questions #2 (effects of treatment) and #3 (effects of screening), we will use the Cochrane Risk of Bias tool [39], which includes assessments of six possible sources of bias related to randomization sequence generation; allocation concealment; blinding of participants, personnel, and outcome assessors; incomplete outcome data; selective outcome data; and other sources of bias. In addition to these domains, based on recent recommendations regarding potential bias due to financial conflicts of interest [40,41], we will code two further domains, for pharmaceutical industry funding, and author-industry financial ties or employment by industry.

### Synthesis of included studies

The decision as to whether data can be synthesized using meta-analysis will be determined based on search results. Based on the studies included in the 2009 United States AHRQ systematic review [1] and a rough preliminary search of MEDLINE conducted in October 2011, we expect that there will be substantial heterogeneity between diagnostic accuracy studies with respect to assessment timing, criterion standards, screening instruments, and scoring thresholds. Most studies have used population-specific cutoff thresholds based on receiver operator characteristic curves, which yields overly optimistic estimates of screening accuracy that do not replicate consistently [42]. Similarly, for studies of MDD treatment, there appears to be substantial heterogeneity in the nature of interventions, outcome measures, and length of follow-up. Thus, due to clinical heterogeneity, it appears that data pooling will likely not be appropriate and that a systematic review, rather than a meta-analysis, will be conducted. If, following the full literature review, we find that meta-analytic data pooling is feasible, then we will use appropriate models that have been used previously in meta-analyses of diagnostic accuracy [43], screening effectiveness [44], and treatment outcomes from depression care interventions [45,46].

## Discussion

Depression is a chronic and disabling condition that is the leading global cause of life-years lived with disability and the fourth leading cause of disability-adjusted life-years, which takes into account premature mortality [47]. Depression during childhood and adolescence can have a devastating impact, both because of its effect on important childhood and adolescence outcomes and because it is a robust predictor of ongoing mental health problems into adulthood [1,15,17-19].

It is crucial that optimal depression prevention and care programs be developed and implemented to reduce the burden of suffering from childhood and adolescent depression. Universal depression screening has been recommended as a solution. Guidelines and recommendations, however, are sometimes made without full consideration of evidence or clinical practice realities [48]. Current guidelines for the management of childhood and adolescent depression do not all agree about whether universal screening should be conducted [2-4], and depression screening among children and adolescents has been implemented in some settings without evidence of benefit [26-32].

Although screening is sometimes portrayed as the only alternative to simply ignoring depression [49], researchers have accumulated evidence suggesting that patients may benefit more by investing resources into improving programs to better manage depression rather than seeking to identify otherwise unidentified patients, many of whom have lesser levels of symptomatology, and adding them to an already poorly functioning depression care system [50,51]. Indeed, Gilbody *et al*. found that screening does not appear to be a necessary component of effective collaborative care programs for depression in primary care [46]. At this juncture, we know very little about potential benefits of depression screening in childhood and adolescence versus potential harms. A real danger is that implementation of routine screening without evidence of how this is best done or whether it will benefit patients could result in overtreatment of depression due to the prescription of antidepressants based on positive screens without follow-up diagnostic interviews on the one hand, and the continued inadequate treatment of children and adolescents with MDD on the other because resources are consumed by attempting to find new cases rather than providing adequate treatment to children and adolescents who are otherwise identified.

The proposed systematic review will determine whether there is sufficient evidence to support screening for depression in childhood and adolescence. The conclusions drawn from this systematic review, once disseminated to policy-makers, health care providers, and researchers, will allow decisions to be made about whether screening programs are likely to benefit children and adolescents.

## Abbreviations

AHRQ: Agency for Healthcare Research and Quality; CTFPHC: Canadian Task Force on Preventive Health Care; DSM: Diagnostic and Statistical Manual; ICD: International Classification of Diseases; MDD: Major depressive disorder; NICE: National Institute for Health and Clinical Excellence; RCT: Randomized controlled trial; SNRI: Serotonin-norepinephrine reuptake inhibitor; SSRI: Selective serotonin reuptake inhibitor; USPSTF: United States Preventive Services Task Force.

## Competing interests

The authors declare that they have no competing interests.

## Authors’ contributions

BDT conceived and designed the systematic review protocol. BDT and MR drafted the protocol. LAK consulted on the search strategy and developed the search terms included in the protocol. All authors revised the protocol critically and read and approved the final manuscript.

## Supplementary Material

Additional file 1 Relevant systematic reviews and meta-analyses. Click here for file

Additional file 2 Search strategies. Click here for file

Additional file 3 Coding manual. Click here for file

Additional file 4 Variables included in data extraction form. Click here for file
