# Supplementary material for: Depression screening and mental health outcomes in children and adolescents: a systematic review protocol
Source: Syst Rev. 2012 Nov 24;1:58. doi: 10.1186/2046-4053-1-58 (PMC3563607; doi:10.1186/2046-4053-1-58)
Supplement: Additional file 1 — Relevant systematic reviews and meta-analyses. [file 2046-4053-1-58-S1.docx]

**APPENDIX 1: Relevant Systematic Reviews and Meta-Analyses**

**Multi-level (Prevalence, Detection, Screening, Treatment): Children and Adolescents**

1. Williams SB, O’Conner EA, Eder M, Whitlock EP. Screening for child and adolescent depression in primary care settings: A systematic evidence review for the US Preventative Services Task Force. *Pediatrics*. 2009;123:e716-e735.

**Prevalence of Depression in Children and Adolescents**

1. Costello EJ, Alaatin E, Angold A. Is there an epidemic of child and adolescent depression? *J Child Psychol Psychiatry*. 2006: 47;12:1263–1271.
2. Fleming JE, Offord DR. Epidemiology of childhood depressive disorders: A critical review. *Journal of the American Academy of Child & Adolescent Psychiatry.* 1990;29:571-580.

**Detection of Depression in Children and Adolescents**

1. Brooks SJ, Kutcher S. Diagnosis and measurement of adolescent depression: A review of commonly utilized instruments. *Journal of Child and Adolescent Psychopharmacology*. 2001;11:341-376.
2. Zuckerbrot RA, Jensen PS. Improving recognition of adolescent depression in primary care. *Archives of Pediatrics & Adolescent Medicine*. 2006;160:694-704.

**Treatment of Depression in Children and Adolescents**

1. Dubicka B, Elvins R, Roberts C, Chick G, Wilkinson P, Goodyer IM. Combined treatment with cognitive–behavioural therapy in adolescent depression: meta-analysis. [*British Journal of Psychiatry.*](http://www.ncbi.nlm.nih.gov/pubmed/21119148) 2010;197:433-40.
2. [Gentile S](http://www.ncbi.nlm.nih.gov/pubmed?term=%22Gentile%20S%22%5BAuthor%5D). Antidepressant use in children and adolescents diagnosed with major depressive disorder: what can we learn from published data? *Reviews on Recent Clinical Trials.* 2010;5:63-75.
3. Maalouf, FT, Brent DA. Pharmacotherapy and psychotherapy of pediatric depression. *Expert Opinion on Pharmacotherapy*. 2010;11:2129-2140.
4. [Richardson T](http://www.ncbi.nlm.nih.gov/pubmed?term=%22Richardson%20T%22%5BAuthor%5D), [Stallard P](http://www.ncbi.nlm.nih.gov/pubmed?term=%22Stallard%20P%22%5BAuthor%5D), [Velleman S](http://www.ncbi.nlm.nih.gov/pubmed?term=%22Velleman%20S%22%5BAuthor%5D). Computerised cognitive behavioural therapy for the prevention and treatment of depression and anxiety in children and adolescents: a systematic review. [*Clinical Child and Family Psychology Review*.](http://www.ncbi.nlm.nih.gov/pubmed/20532980) 2010;13:275-90.
5. Tsapakis EM, Soldani F, Tondo L, Baldessarini RJ. Efficacy of antidepressants in juvenile depression: meta-analysis. *British Journal of Psychiatry*. 2008;193:10-7.
6. Usala T, Clavenna A, Zuddas A, Bonati M. Randomised controlled trials of selective serotonin reuptake inhibitors in treating depression in children and adolescents: a systematic review and meta-analysis. *European Neuropsychopharmacology*. 2008;18:62-73
7. Hetrick SE, Merry SN, McKenzie J, Sindahl P, Proctor M. Selective serotonin reuptake inhibitors (SSRIs) for depressive disorders in children and adolescents. *Cochrane Database of Systematic Reviews.* 2007;Issue 3:CD004851.
8. Weisz JR, McCarty CA, Valeri SM . Effects of psychotherapy for depression in children and adolescents: A meta-analysis. *Psychological Bulletin.* 2006;132: 132-149.
9. Compton SN, March JS, Brent D, et al. Cognitive-behavioral psychotherapy for anxiety and depressive disorders in children and adolescents: an evidence-based medicine review. *Journal of the American Academy of Child & Adolescent Psychiatry*. 2004;43:930-959.
10. Courtney DB. Selective serotonin reuptake inhibitor and venlafaxine use in children and adolescents with major depressive disorder: a systematic review of published randomized controlled trials. *Canadian Journal of Psychiatry*. 2004;49:557-63.
11. Whittington CJ, Kendall T, Fonagy P, Cottrell D, Cotgrove A, Boddington E. Selective serotonin reuptake inhibitors in childhood depression: systematic review of published versus unpublished data *Lancet*. 2004; 1341-1345.
12. O’Connell D, Heathcote D, Henry D, Hazell P. Tricyclic drugs for depression in children and adolescents. *Cochrane Database of Systematic Reviews*. 2002; Issue 2:CD002317.
13. Harrington R, Whittaker J, Shoebridge P, Campbell F. Systematic review of cognitive behavioral therapies in childhood and adolescent depressive disorder. *British Medical Journal.* 1998;316:1559-63.
14. Reinecke, MA, Ryan, NE, & DuBois, DL. Cognitive–behavioral therapy of depression and depressive symptoms during adolescence: A review and meta-analysis. *Journal of the American Academy of Child & Adolescent Psychiatry.* 1998;37:26–34.

**Screening for Depression in Children and Adolescents**

1. Williams SB, O’Conner EA, Eder M, Whitlock EP. Screening for child and adolescent depression in primary care settings: A systematic evidence review for the US Preventative Services Task Force. *Pediatrics*. 2009;123:e716-e735.

**Harms Related to Treating Depression in Children and Adolescents**

1. Bridge JA, Iyengar S, Salary CB, et al. Clinical response and risk for reported suicidal ideation and suicide attempts in pediatric antidepressant treatment. *JAMA*. 2007;297:1683-1696.
2. Hammad TA, Laughren T, Racoosin J. Suicidality in pediatric patients treated with antidepressant drugs. *Archives of General Psychiatry.* 2006;63:332-339.
3. Sharp SC, Hellings JA. Efficacy and safety of selective serotonin reuptake inhibitors in the treatment of depression in children and adolescents: practitioner review. *Clinical Drug Investigation.* 2006;26:247-255.
4. Tamar D, Wohlfarth BJ, van Zwieten FJ, et al. Antidepressants use in children and adolescents and the risk of suicide. [*European Neuropsychopharmacology*](http://www.sciencedirect.com/science/journal/0924977X). 2006;16:79-83.
5. Fergusson D, Doucette S, Glass KC, et al. Association between suicide attempts and selective serotonin reuptake inhibitors: systematic review of randomised controlled trials. *British Medical Journal.* 2005;330:396.
