# Supplementary material for: Depression screening and mental health outcomes in children and adolescents: a systematic review protocol
Source: Syst Rev. 2012 Nov 24;1:58. doi: 10.1186/2046-4053-1-58 (PMC3563607; doi:10.1186/2046-4053-1-58)
Supplement: Additional file 2 — Search strategies. [file 2046-4053-1-58-S2.docx]

**APPENDIX 2: Search Strategies**

**Search Strategies for Key Question #1**

**MEDLINE**

1. Depression/

2. Depressive Disorder/

3. Depressive Disorder, Major/

4. depress*.ti.

5. or/1-4

6. exp Psychological Tests/

7. exp Psychiatric Status Rating Scales/

8. *Questionnaires/

9. Self-report/

10. Mass Screening/

11. case finding*.ti,ab.

12. casefinding*.ti,ab.

13. ((screen* or evaluat* or assess* or diagnos* or rating or rate or measur*) adj5 (index or indices or score* or scoring or test* or instrument* or inventor* or battery or batteries or tool* or scale* or checklist* or schedule*)).ti,ab.

14. ("mood and feelings questionnaire*" or MFQ or SMFQ or MFQ-C).ti,ab.

15. reynold* child* depression.ti,ab.

16. Reynold* adolesc* depression.ti,ab.

17. kutcher* adolesc*.ti,ab.

18. "depression scale for child*".ti,ab.

19. (Children's Depression Inventory or CDI).ti,ab.

20. (Brief Patient Health Questionnaire-9 or PHQ-9).ti,ab.

21. (Brief Patient Health Questionnaire-2 or PHQ-2).ti,ab.

22. (Brief Patient Health Questionnaire-A or PHQ-A).ti,ab.

23. (Center for Epidemiologic Studies Depression Scale or CES-D).ti,ab.

24. (Beck Depression Inventory or BDI or BDI-PC).ti,ab.

25. (Hopkins Symptom Checklist or HSCL or HSCL-10).ti,ab.

26. ("Strengths and Difficulties Questionnaire" or SDQ).ti,ab.

27. (Youth Self Report or YSR).ti,ab.

28. DesTeen.ti,ab.

29. or/6-28

30. 5 and 29

31. limit 30 to validation studies

32. (valid* or predict* or identif* or detect*).ti.

33. "Sensitivity and Specificity"/

34. "Predictive Value of Tests"/

35. (cut?off* or accura* or gold standard or reference standard).ab.

36. or/32-35

37. 30 and 36

38. 31 or 37

39. exp Child/

40. child.mp.

41. exp Pediatrics/

42. pediatric*.mp.

43. paediatric*.mp.

44. (boy or boys).mp.

45. girl*.mp.

46. (kid or kids).mp.

47. school?age*.mp.

48. juvenil*.mp.

49. under?age*.mp.

50. teen*.mp.

51. minor*.mp.

52. pubescen*.mp.

53. adolescen*.mp.

54. (youth or youths).mp.

55. child*.jw.

56. pediatric*.jw.

57. paediatric*.jw.

58. adolescen*.jw.

59. or/39-58

60. 38 and 59

61. limit 60 to yr="2006 -Current"

62. Remove duplicates from 61

**MEDLINE In-Process**

1. MDD.ti,ab.

2. depress*.ti,ab.

3. 1 or 2 [Depression]

4. Psych* Test*.ti,ab.

5. case finding*.ti,ab.

6. casefinding*.ti,ab.

7. ((screen* or evaluat* or assess* or diagnos* or rating or rate or measur*) adj5 (index or indices or score* or scoring or test* or instrument* or inventor* or battery or batteries or tool* or scale* or checklist* or schedule*)).ti,ab.

8. ("mood and feelings questionnaire*" or MFQ or SMFQ or MFQ-C).ti,ab.

9. reynold* child* depression.ti,ab.

10. Reynold* adolesc* depression.ti,ab.

11. kutcher* adolesc*.ti,ab.

12. "depression scale for child*".ti,ab.

13. (Children's Depression Inventory or CDI).ti,ab.

14. (Brief Patient Health Questionnaire-9 or PHQ-9).ti,ab.

15. (Brief Patient Health Questionnaire-2 or PHQ-2).ti,ab.

16. (Brief Patient Health Questionnaire-A or PHQ-A).ti,ab.

17. (Center for Epidemiologic Studies Depression Scale or CES-D).ti,ab.

18. (Beck Depression Inventory or BDI or BDI-PC).ti,ab.

19. (Hopkins Symptom Checklist or HSCL or HSCL-10).ti,ab.

20. ("Strengths and Difficulties Questionnaire" or SDQ).ti,ab.

21. (Youth Self Report or YSR).ti,ab.

22. DesTeen.ti,ab.

23. or/4-22 [Screening]

24. (valid* or predict* or identif* or diagnos* or accura* or detect* or cut?off* or gold standard).ti,ab.

25. child.mp.

26. pediatric*.mp.

27. paediatric*.mp.

28. (boy or boys).mp.

29. girl*.mp.

30. (kid or kids).mp.

31. school?age*.mp.

32. juvenil*.mp.

33. under?age*.mp.

34. teen*.mp.

35. minor*.mp.

36. pubescen*.mp.

37. adolescen*.mp.

38. (youth or youths).mp.

39. child*.jw.

40. pediatric*.jw.

41. paediatric*.jw.

42. adolescen*.jw.

43. or/25-42

44. 3 and 23 and 24 and 43

**HaPI**

1. MDD.ti,ab.

2. depress*.ti,ab.

3. ("mood and feelings questionnaire*" or MFQ or SMFQ or MFQ-C).ti,ab.

4. reynold* child* depression.ti,ab.

5. Reynold* adolesc* depression.ti,ab.

6. kutcher* adolesc*.ti,ab.

7. "depression scale for child*".ti,ab.

8. (Children's Depression Inventory or CDI).ti,ab.

9. (Brief Patient Health Questionnaire-9 or PHQ-9).ti,ab.

10. (Brief Patient Health Questionnaire-2 or PHQ-2).ti,ab.

11. (Brief Patient Health Questionnaire-A or PHQ-A).ti,ab.

12. (Center for Epidemiologic Studies Depression Scale or CES-D).ti,ab.

13. (Beck Depression Inventory or BDI or BDI-PC).ti,ab.

14. (Hopkins Symptom Checklist or HSCL or HSCL-10).ti,ab.

15. ("Strengths and Difficulties Questionnaire" or SDQ).ti,ab.

16. (Youth Self Report or YSR).ti,ab.

17. DesTeen.ti,ab.

18. or/1-17

19. (valid* or predict* or identif* or diagnos* or accura* or detect* or cut?off* or gold standard).ti,ab.

20. child.mp.

21. pediatric*.mp.

22. paediatric*.mp.

23. (boy or boys).mp.

24. girl*.mp.

25. (kid or kids).mp.

26. school?age*.mp.

27. juvenil*.mp.

28. under?age*.mp.

29. teen*.mp.

30. minor*.mp.

31. pubescen*.mp.

32. adolescen*.mp.

33. (youth or youths).mp.

34. child*.jw.

35. pediatric*.jw.

36. paediatric*.jw.

37. adolescen*.jw.

38. or/20-37

39. 18 and 19 and 38

40. limit 39 to yr=”2006-Current”

**EMBASE**

1. Depression/

2. Depressive Disorder, Major/

3. depress*.ti.

4. or/1-3

5. exp Psychologic Test/

6. Psychiatric Diagnosis/

7. *Questionnaire/

8. Self-report/

9. Mass Screening/

10. case finding*.ti,ab.

11. casefinding*.ti,ab.

12. ((screen* or evaluat* or assess* or diagnos* or rating or rate or measur*) adj5 (index or indices or score* or scoring or test* or instrument* or inventor* or battery or batteries or tool* or scale* or checklist* or schedule*)).ti,ab.

13. ("mood and feelings questionnaire*" or MFQ or SMFQ or MFQ-C).ti,ab.

14. reynold* child* depression.ti,ab.

15. Reynold* adolesc* depression.ti,ab.

16. kutcher* adolesc*.ti,ab.

17. "depression scale for child*".ti,ab.

18. (Children's Depression Inventory or CDI).ti,ab.

19. (Brief Patient Health Questionnaire-9 or PHQ-9).ti,ab.

20. (Brief Patient Health Questionnaire-2 or PHQ-2).ti,ab.

21. (Brief Patient Health Questionnaire-A or PHQ-A).ti,ab.

22. (Center for Epidemiologic Studies Depression Scale or CES-D).ti,ab.

23. (Beck Depression Inventory or BDI or BDI-PC).ti,ab.

24. (Hopkins Symptom Checklist or HSCL or HSCL-10).ti,ab.

25. ("Strengths and Difficulties Questionnaire" or SDQ).ti,ab.

26. (Youth Self Report or YSR).ti,ab.

27. DesTeen.ti,ab.

28. or/5-27

29. (valid* or predict* or identif* or detect*).ti.

30. "Sensitivity and Specificity"/

31. Predictive Value/

32. (cut?off* or accura* or gold standard or reference standard).ab.

33. Validity/

34. or/29-33

35. exp Child/

36. child.mp.

37. Pediatrics/

38. Child Psychology/

39. Child Psychiatry/

40. pediatric*.mp.

41. paediatric*.mp.

42. (boy or boys).mp.

43. girl*.mp.

44. (kid or kids).mp.

45. school?age*.mp.

46. juvenil*.mp.

47. under?age*.mp.

48. teen*.mp.

49. minor*.mp.

50. pubescen*.mp.

51. adolescen*.mp.

52. (youth or youths).mp.

53. child*.jx.

54. pediatric*.jx.

55. paediatric*.jx.

56. adolescen*.jx.

57. or/35-56

58. 4 and 28 and 34 and 57

59. limit 58 to yr="2006 -Current"

60. Remove duplicates from 59

**PsycINFO**

1. "Depression (Emotion)"/

2. Major Depression/

3. depress*.ti.

4. or/1-3

5. ("mood and feelings questionnaire*" or MFQ or SMFQ or MFQ-C).ti,ab.

6. reynold* child* depression.ti,ab.

7. Reynold* adolesc* depression.ti,ab.

8. kutcher* adolesc*.ti,ab.

9. "depression scale for child*".ti,ab.

10. (Children's Depression Inventory or CDI).ti,ab.

11. (Brief Patient Health Questionnaire-9 or PHQ-9).ti,ab.

12. (Brief Patient Health Questionnaire-2 or PHQ-2).ti,ab.

13. (Brief Patient Health Questionnaire-A or PHQ-A).ti,ab.

14. (Center for Epidemiologic Studies Depression Scale or CES-D).ti,ab.

15. (Beck Depression Inventory or BDI or BDI-PC).ti,ab.

16. (Hopkins Symptom Checklist or HSCL or HSCL-10).ti,ab.

17. ("Strengths and Difficulties Questionnaire" or SDQ).ti,ab.

18. (Youth Self Report or YSR).ti,ab.

19. DesTeen.ti,ab.

20. Psychological Assessment/

21. Testing/

22. Psychodiagnosis/

23. Psychiatric Evaluation/

24. Screening/

25. Screening Tests/

26. Health Screening/

27. Rating Scales/

28. Self Report/

29. Self Evaluation/

30. Questionnaires/

31. Inventories/

32. Symptom Checklists/

33. case?finding*.ti,ab.

34. ((screen* or evaluat* or assess* or diagnos* or rating or rate or measur*) adj5 (index or indices or score* or scoring or test* or instrument* or inventor* or battery or batteries or tool* or scale* or checklist* or schedule*)).ti,ab.

35. or/5-34

36. (valid* or predict* or identif* or diagnos* or accura* or detect* or cut?off* or gold standard).ti,ab.

37. Prediction/

38. Test Validity/

39. Misdiagnosis/

40. Test Interpretation/

41. Cutting Scores/

42. or/36-41

43. child.mp.

44. Pediatrics/

45. Child Psychology/

46. Child Psychiatry/

47. pediatric*.mp.

48. paediatric*.mp.

49. (boy or boys).mp.

50. girl*.mp.

51. (kid or kids).mp.

52. school?age*.mp.

53. juvenil*.mp.

54. under?age*.mp.

55. teen*.mp.

56. minor*.mp.

57. pubescen*.mp.

58. adolescen*.mp.

59. (youth or youths).mp.

60. child*.jx.

61. pediatric*.jx.

62. paediatric*.jx.

63. adolescen*.jx.

64. or/43-63

65. 4 and 35 and 42 and 64

66. limit 65 to yr="2006 -Current"

67. remove duplicates from 66

**LILACS**

(ti:Depress$ OR mh:depression OR mh:”depressive disorder” OR mh:”depressive disorder, major”) AND (test$ OR instrument$ OR measure$ OR inventor$ OR scale$ OR evaluat$ OR screen$ OR assess$ OR rating$ OR rate$ OR score$ OR checklist$ OR batter$ OR score$ OR scoring OR diagnos$) AND (child$ OR teen$ OR adolescen$ OR youth$ OR pediatric$ OR paediatric$) AND (valid$ OR predict$ OR accura$ OR detect$ OR cut off$ OR gold standard$ OR identif$ OR diagnos$) AND (da:2006$ OR da:2007$ or da:2008$ OR da:2009$ or da:2010$ OR da:2011$ OR da:2012$)

**Search Strategies for Key Question #2**

**MEDLINE**

1. Depression/

2. Depressive Disorder/

3. Depressive Disorder, Major/

4. depress*.ti.

5. or/1-4

6. Antidepressive Agents/

7. Antidepressive Agents, Second-Generation/

8. Serotonin Uptake Inhibitors/

9. Fluoxetine/

10. (fluoxetine or prozac or rapiflux or sarafem or selfemra or fontex or zacton or lovan).ti,ab.

11. Sertraline/

12. (sertraline or zoloft or lustral).ti,ab.

13. Paroxetine/

14. (paroxetine or paxil or pexevra or aropax or seroxat or sereupin).ti,ab.

15. Citalopram/

16. (citalopram or celexa or cipramil).ti,ab.

17. Escitalopram/

18. (escitalopram or lexapro or cipralex or seroplex or lexamil or lexam or entact).ti,ab.

19. Fluvoxamine/

20. (fluvoxamine or luvox).ti,ab.

21. Combined Modality Therapy/

22. SSRI.ti,ab.

23. SSRIs.ti,ab.

24. serotonin uptake inhibitor*.ti,ab.

25. serotonin reuptake inhibitor*.ti,ab.

26. antidepress*.ti,ab.

27. anti-depress*.ti,ab.

28. pharmacologic*.ti.

29. pharmacotherap*.ti.

30. exp Psychotherapy/

31. cognitive behavio?ral.ti,ab.

32. CBT.ti,ab.

33. MBCT.ti,ab.

34. mindfulness.ti,ab.

35. family support.ti,ab.

36. family therapy.ti,ab.

37. parent* education.ti,ab.

38. Parents/ed

39. Counseling/

40. Directive Counseling/

41. counsel.ti,ab.

42. group therapy.ti,ab.

43. psychodynamic therapy.ti,ab.

44. future directed therapy.ti,ab.

45. (cognitive adj (therap* or treatment* or intervention*)).ti,ab.

46. (behavio* adj (therap* or treatment* or intervention*)).ti,ab.

47. psychotherap*.ti,ab.

48. interpersonal therap*.ti,ab.

49. interpersonal intervention*.ti,ab.

50. IPT-AST.ti,ab.

51. IPT-A.ti,ab.

52. (acceptance adj commitment therapy).ti,ab.

53. psychoeducation.ti,ab.

54. psycho education.ti,ab.

55. dynamic therapy.ti,ab.

56. (brief adj2 therap*).ti.

57. Therapy, Computer-Assisted/

58. ((internet or online or on-line or web) adj2 (therap* or treatment* or intervention*)).ti,ab.

59. Self-Care/

60. Self-Help Groups/

61. self-help.ti,ab.

62. wait* list control.ti,ab.

63. treatment as usual.ti,ab.

64. Problem Solving/

65. problem solving.ti,ab.

66. behavio* activation therapy.ti,ab.

67. physical activit*.ti,ab.

68. or/6-67

69. limit 68 to yr="2006 -Current"

70. SNRI.ti,ab.

71. SNRIs.ti,ab.

72. norepinephrine reuptake inhibitor*.ti,ab.

73. (venlafaxine or effexor or efexor).ti,ab.

74. (duloxetine or cymbalta or ariclaim or xeristar or yentreve or duzela).ti,ab.

75. (desvenlafaxine or pristiq).ti,ab.

76. Bupropion/

77. (bupropion or wellbutrin or aplenzin or zyban or voxra or budeprion).ti,ab.

78. (mirtazapine or remeron or avanza zispin).ti,ab.

79. Trazodone/

80. (trazodone or desyrel or oleptro or beneficat or deprax or desirel or molipaxin or thombran or trazorel or trialodine or trittico or mesyrel).ti,ab.

81. exp Exercise/

82. "Physical Education and Training"/

83. exercis*.ti,ab.

84. physical activit*.ti,ab.

85. or/70-84

86. 69 or 85

87. Child/

88. child.mp.

89. Minors/

90. Pediatrics/

91. pediatric*.mp.

92. paediatric*.mp.

93. (boy or boys).mp.

94. girl*.mp.

95. (kid or kids).mp.

96. school?age*.mp.

97. juvenil*.mp.

98. under?age*.mp.

99. Adolescent/

100. teen*.mp.

101. pubescen*.mp.

102. adolescen*.mp.

103. (youth or youths).mp.

104. child*.jw.

105. pediatric*.jw.

106. paediatric*.jw.

107. adolescen*.jw.

108. or/87-107

109. Randomized controlled trial.pt.

110. controlled clinical trial.pt.

111. randomized.ab.

112. placebo.ab.

113. clinical trials as topic.sh.

114. randomly.ab.

115. trial.ti.

116. or/109-115

117. exp animals/ not humans.sh.

118. 116 not 117

119. 5 and 86 and 108 and 118

120. remove duplicates from 119

**MEDLINE In-Process**

1. MDD.ti,ab.

2. depress*.ti,ab.

3. 1 or 2

4. (fluoxetine or prozac or rapiflux or sarafem or selfemra or fontex or zacton or lovan).ti,ab.

5. (sertraline or zoloft or lustral).ti,ab.

6. (paroxetine or paxil or pexevra or aropax or seroxat or sereupin).ti,ab.

7. (citalopram or celexa or cipramil).ti,ab.

8. (escitalopram or lexapro or cipralex or seroplex or lexamil or lexam or entact).ti,ab.

9. (fluvoxamine or luvox).ti,ab.

10. (venlafaxine or effexor or efexor).ti,ab.

11. (duloxetine or cymbalta or ariclaim or xeristar or yentreve or duzela).ti,ab.

12. (desvenlafaxine or pristiq).ti,ab.

13. (bupropion or wellbutrin or aplenzin or zyban or voxra or budeprion).ti,ab.

14. (mirtazapine or remeron or avanza zispin).ti,ab.

15. (trazodone or desyrel or oleptro or beneficat or deprax or desirel or molipaxin or thombran or trazorel or trialodine or trittico or mesyrel).ti,ab.

16. ((combined or multimodal) adj (treatment* or intervention* or therap*)).ti,ab.

17. SSRI.ti,ab.

18. SSRIs.ti,ab.

19. SNRI.ti,ab.

20. SNRIs.ti,ab.

21. serotonin uptake inhibitor*.ti,ab.

22. serotonin reuptake inhibitor*.ti,ab.

23. norepinephrine reuptake inhibitor*.ti,ab.

24. antidepress*.ti,ab.

25. anti-depress*.ti,ab.

26. pharmacologic*.ti.

27. pharmacotherap*.ti.

28. cognitive behavio?ral.ti,ab.

29. CBT.ti,ab.

30. MBCT.ti,ab.

31. mindfulness.ti,ab.

32. family support.ti,ab.

33. family therapy.ti,ab.

34. parent* education.ti,ab.

35. counsel.ti,ab.

36. group therapy.ti,ab.

37. psychodynamic therapy.ti,ab.

38. future directed therapy.ti,ab.

39. (cognitive adj (therap* or treatment* or intervention*)).ti,ab.

40. (behavio* adj (therap* or treatment* or intervention*)).ti,ab.

41. psychotherap*.ti,ab.

42. interpersonal therap*.ti,ab.

43. interpersonal intervention*.ti,ab.

44. IPT-AST.ti,ab.

45. IPT-A.ti,ab.

46. (acceptance adj commitment therapy).ti,ab.

47. psychoeducation.ti,ab.

48. psycho education.ti,ab.

49. dynamic therapy.ti,ab.

50. (brief adj2 therap*).ti.

51. computer assisted therap*.ti,ab.

52. ((internet or online or on-line or web) adj2 (therap* or treatment* or intervention*)).ti,ab.

53. self-help.ti,ab.

54. wait* list control.ti,ab.

55. treatment as usual.ti,ab.

56. problem solving.ti,ab.

57. behavio* activation therapy.ti,ab.

58. exercis*.ti,ab.

59. physical activit*.ti,ab.

60. or/4-59

61. child.mp.

62. pediatric*.mp.

63. paediatric*.mp.

64. (boy or boys).mp.

65. girl*.mp.

66. (kid or kids).mp.

67. school?age*.mp.

68. juvenil*.mp.

69. under?age*.mp.

70. teen*.mp.

71. pubescen*.mp.

72. adolescen*.mp.

73. (youth or youths).mp.

74. child*.jw.

75. pediatric*.jw.

76. paediatric*.jw.

77. adolescen*.jw.

78. or/61-77

79. (random* or placebo* or trial*).mp.

80. 3 and 60 and 78 and 79

**EMBASE**

1. Depressive Disorder/

2. Depressive Disorder, Major/

3. depress*.ti.

4. or/1-3

5. Antidepressive Agents/

6. Antidepressive Agents, Second-Generation/

7. Serotonin Uptake Inhibitors/

8. Fluoxetine/

9. (fluoxetine or prozac or rapiflux or sarafem or selfemra or fontex or zacton or lovan).ti,ab.

10. Sertraline/

11. (sertraline or zoloft or lustral).ti,ab.

12. Paroxetine/

13. (paroxetine or paxil or pexevra or aropax or seroxat or sereupin).ti,ab.

14. Citalopram/

15. (citalopram or celexa or cipramil).ti,ab.

16. Escitalopram/

17. (escitalopram or lexapro or cipralex or seroplex or lexamil or lexam or entact).ti,ab.

18. Fluvoxamine/

19. (fluvoxamine or luvox).ti,ab.

20. Combined Modality Therapy/

21. SSRI.ti,ab.

22. SSRIs.ti,ab.

23. serotonin uptake inhibitor*.ti,ab.

24. serotonin reuptake inhibitor*.ti,ab.

25. antidepress*.ti,ab.

26. anti-depress*.ti,ab.

27. pharmacologic*.ti.

28. pharmacotherap*.ti.

29. exp Psychotherapy/

30. cognitive behavio?ral.ti,ab.

31. CBT.ti,ab.

32. MBCT.ti,ab.

33. mindfulness.ti,ab.

34. family support.ti,ab.

35. family therapy.ti,ab.

36. parent* education.ti,ab.

37. Parents/ed

38. Counseling/

39. Directive Counseling/

40. counsel.ti,ab.

41. group therapy.ti,ab.

42. psychodynamic therapy.ti,ab.

43. future directed therapy.ti,ab.

44. (cognitive adj (therap* or treatment* or intervention*)).ti,ab.

45. (behavio* adj (therap* or treatment* or intervention*)).ti,ab.

46. psychotherap*.ti,ab.

47. interpersonal therap*.ti,ab.

48. interpersonal intervention*.ti,ab.

49. IPT-AST.ti,ab.

50. IPT-A.ti,ab.

51. (acceptance adj commitment therapy).ti,ab.

52. psychoeducation.ti,ab.

53. psycho education.ti,ab.

54. dynamic therapy.ti,ab.

55. (brief adj2 therap*).ti.

56. Therapy, Computer-Assisted/

57. ((internet or online or on-line or web) adj2 (therap* or treatment* or intervention*)).ti,ab.

58. Self-Care/

59. Self-Help Groups/

60. self-help.ti,ab.

61. wait* list control.ti,ab.

62. treatment as usual.ti,ab.

63. Problem Solving/

64. problem solving.ti,ab.

65. behavio* activation therapy.ti,ab.

66. or/5-65

67. limit 66 to yr="2006 -Current"

68. Noradrenaline Uptake Inhibitor/

69. norepinephrine reuptake inhibitor*.ti,ab.

70. SNRI.ti,ab.

71. SNRIs.ti,ab.

72. (venlafaxine or effexor or efexor).ti,ab.

73. (duloxetine or cymbalta or ariclaim or xeristar or yentreve or duzela).ti,ab.

74. (desvenlafaxine or pristiq).ti,ab.

75. Bupropion/

76. (bupropion or wellbutrin or aplenzin or zyban or voxra or budeprion).ti,ab.

77. (mirtazapine or remeron or avanza zispin).ti,ab.

78. Trazodone/

79. (trazodone or desyrel or oleptro or beneficat or deprax or desirel or molipaxin or thombran or trazorel or trialodine or trittico or mesyrel).ti,ab.

80. exp Exercise/

81. Exercise Therapy/

82. Exercise Movement/

83. "Physical Education and Training"/

84. exercis*.ti,ab.

85. physical activit*.ti,ab.

86. or/68-85

87. 67 or 86

88. Child/

89. child.mp.

90. Minors/

91. Pediatrics/

92. pediatric*.mp.

93. paediatric*.mp.

94. (boy or boys).mp.

95. girl*.mp.

96. (kid or kids).mp.

97. school?age*.mp.

98. juvenil*.mp.

99. under?age*.mp.

100. Adolescent/

101. teen*.mp.

102. pubescen*.mp.

103. adolescen*.mp.

104. (youth or youths).mp.

105. child*.jx.

106. pediatric*.jx.

107. paediatric*.jx.

108. adolescen*.jx.

109. or/88-108

110. random*.tw. or placebo*.mp. or double-blind*.tw.

111. 4 and 87 and 109 and 110

112. remove duplicates from 111

**PsycINFO**

1. "Depression (Emotion)"/

2. Major Depression/

3. depress*.ti.

4. or/1-3

5. Antidepressant Drugs/

6. Serotonin Reuptake Inhibitors/

7. Fluoxetine/

8. (fluoxetine or prozac or rapiflux or sarafem or selfemra or fontex or zacton or lovan).ti,ab.

9. Sertraline/

10. (sertraline or zoloft or lustral).ti,ab.

11. Paroxetine/

12. (paroxetine or paxil or pexevra or aropax or seroxat or sereupin).ti,ab.

13. Citalopram/

14. (citalopram or celexa or cipramil).ti,ab.

15. Escitalopram/

16. (escitalopram or lexapro or cipralex or seroplex or lexamil or lexam or entact).ti,ab.

17. Fluvoxamine/

18. (fluvoxamine or luvox).ti,ab.

19. Multimodal Treatment Approach/

20. SSRI.ti,ab.

21. SSRIs.ti,ab.

22. serotonin uptake inhibitor*.ti,ab.

23. serotonin reuptake inhibitor*.ti,ab.

24. antidepress*.ti,ab.

25. anti-depress*.ti,ab.

26. pharmacologic*.ti.

27. pharmacotherap*.ti.

28. Psychotherapy/

29. Individual Psychotherapy/

30. cognitive behavio?ral.ti,ab.

31. CBT.ti,ab.

32. MBCT.ti,ab.

33. mindfulness.ti,ab.

34. family support.ti,ab.

35. family therapy.ti,ab.

36. parent* education.ti,ab.

37. Parent Training/

38. Counseling/

39. counsel.ti,ab.

40. group therapy.ti,ab.

41. psychodynamic therapy.ti,ab.

42. future directed therapy.ti,ab.

43. (cognitive adj (therap* or treatment* or intervention*)).ti,ab.

44. (behavio* adj (therap* or treatment* or intervention*)).ti,ab.

45. psychotherap*.ti,ab.

46. interpersonal therap*.ti,ab.

47. interpersonal intervention*.ti,ab.

48. IPT-AST.ti,ab.

49. IPT-A.ti,ab.

50. (acceptance adj commitment therapy).ti,ab.

51. psychoeducation.ti,ab.

52. psycho education.ti,ab.

53. dynamic therapy.ti,ab.

54. (brief adj2 therap*).ti.

55. Computer Assisted Therapy/

56. ((internet or online or on-line or web) adj2 (therap* or treatment* or intervention*)).ti,ab.

57. Self-Care/

58. Self-Help Techniques/

59. self-help.ti,ab.

60. wait* list control.ti,ab.

61. treatment as usual.ti,ab.

62. Problem Solving/

63. problem solving.ti,ab.

64. behavio* activation therapy.ti,ab.

65. or/5-64

66. limit 65 to yr="2006 -Current"

67. Serotonin Norepinephrine Reuptake Inhibitors/

68. norepinephrine reuptake inhibitor*.ti,ab.

69. SNRI.ti,ab.

70. SNRIs.ti,ab.

71. Venlafaxine/

72. (venlafaxine or effexor or efexor).ti,ab.

73. (duloxetine or cymbalta or ariclaim or xeristar or yentreve or duzela).ti,ab.

74. (desvenlafaxine or pristiq).ti,ab.

75. Bupropion/

76. (bupropion or wellbutrin or aplenzin or zyban or voxra or budeprion).ti,ab.

77. (mirtazapine or remeron or avanza zispin).ti,ab.

78. Trazodone/

79. (trazodone or desyrel or oleptro or beneficat or deprax or desirel or molipaxin or thombran or trazorel or trialodine or trittico or mesyrel).ti,ab.

80. exp Exercise/

81. exercis*.ti,ab.

82. physical activit*.ti,ab.

83. or/67-82

84. 66 or 83

85. Child Psychology/

86. child.mp.

87. Child Psychiatry/

88. Pediatrics/

89. pediatric*.mp.

90. paediatric*.mp.

91. (boy or boys).mp.

92. girl*.mp.

93. (kid or kids).mp.

94. school?age*.mp.

95. juvenil*.mp.

96. under?age*.mp.

97. teen*.mp.

98. pubescen*.mp.

99. adolescen*.mp.

100. (youth or youths).mp.

101. child*.jx.

102. pediatric*.jx.

103. paediatric*.jx.

104. adolescen*.jx.

105. or/85-104

106. (control* or random*).tw. or exp treatment/

107. 4 and 84 and 105 and 106

108. remove duplicates from 107

**CENTRAL**

#1 [(treatment* or intervention* or psychotherapy or therap* or pharmacotherap* or drug* or SSRI* or serotonin reuptake inhibitor* or anti-depress* or antidepress* or fluoxetine or sertraline or paroxetine or citalopram or escitalopram or fluvoxamine or counsel*):ti,ab,kw, from 2006 to 2012 in Trials](http://onlinelibrary.wiley.com/o/cochrane/searchHistory?mode=runquery&qnum=1) 112064
#2 [(norepinephrine reuptake inhibitor* or noradrenaline reuptake inhibitor* or SNRI* or venlafaxine or duloxetine or desvenlafaxine or bupropion or mirtazapine or trazodone or exercis*):ti,ab,kw in Trials](http://onlinelibrary.wiley.com/o/cochrane/searchHistory?mode=runquery&qnum=2) 33360
#3 [(depression or depressive or MDD) :ti,ab,kw in Trials](http://onlinelibrary.wiley.com/o/cochrane/searchHistory?mode=runquery&qnum=3) 26926
#4 [(child* or teen* or adolescen* or youth* or boy* or girl* or juvenile* or pediatric* or paediatric*):ti,ab,kw in Trials](http://onlinelibrary.wiley.com/o/cochrane/searchHistory?mode=runquery&qnum=4) 113320
#5 [(#1 OR #2)](http://onlinelibrary.wiley.com/o/cochrane/searchHistory?mode=runquery&qnum=5) 160777
#6 [(#3 AND #4 AND #5)](http://onlinelibrary.wiley.com/o/cochrane/searchHistory?mode=runquery&qnum=6) 1590

**LILACS**

(ti:Depress$ OR mh:depression OR mh:"depressive disorder" OR mh:"depressive disorder, major") AND (psychotherapy OR pharmacotherapy$ OR drug$ OR SSRI$ OR SNRI$ OR anti-depress$ OR antidepress$ OR fluoxetine OR sertraline OR paroxetine OR citalopram OR escitalopram OR fluvoxamine OR venlafaxine OR duloxetine OR desvenlafaxine OR bupropion OR mirtazapine OR trazodone OR counsel$ OR exercis$) AND (child$ OR teen$ OR adolescen$ OR youth$ OR pediatric$ OR paediatric$) AND (da:2006$ OR da:2007$ OR da:2008$ OR da:2009$ OR da:2010$ OR da:2011$ OR da:2012$)
Type of study: Controlled Clinical Trials

**Clinicaltrials.gov**

"Depressive Disorder, Major" | Child | received from 01/01/2006 to 12/31/2012

venlafaxine OR duloxetine OR desvenlafaxine OR bupropion OR mirtazapine OR trazodone OR exercise | Major Depressive Disorder | Child

**International Clinical Trials Registry Platform**

Advanced Search

Depression OR Major Depressive Disorder (in Condition)
Drug OR psychotherapy OR exercise (in Intervention)
Search for clinical trials in children
Recruitment status is ALL
Date of registration is between: 01/01/2006 and 31/12/2012

And

Depression OR Major Depressive Disorder (in Condition)
venlafaxine OR duloxetine OR desvenlafaxine OR bupropion OR mirtazapine OR trazodone OR exercise (in Intervention)
Search for clinical trials in children
Recruitment status is ALL
Date of registration is between: [blank] and 31/12/2012

**Search Strategies for Key Question #3**

**MEDLINE**

1. Depression/

2. Depressive Disorder/

3. Depressive Disorder, Major/

4. depress*.ti.

5. or/1-4

6. Mass Screening/

7. screening.ti.

8. 6 or 7

9. Child/

10. child.mp.

11. Pediatrics/

12. pediatric*.mp.

13. paediatric*.mp.

14. (boy or boys).mp.

15. girl*.mp.

16. (kid or kids).mp.

17. school?age*.mp.

18. juvenil*.mp.

19. under?age*.mp.

20. teen*.mp.

21. pubescen*.mp.

22. Adolescent/

23. adolescen*.mp.

24. (youth or youths).mp.

25. Minors/

26. child*.jw.

27. pediatric*.jw.

28. paediatric*.jw.

29. adolescen*.jw.

30. or/9-29

31. Randomized controlled trial.pt.

32. controlled clinical trial.pt.

33. randomized.ab.

34. placebo.ab.

35. clinical trials as topic.sh.

36. randomly.ab.

37. trial.ti.

38. or/31-37

39. exp animals/ not humans.sh.

40. 38 not 39

41. 5 and 8 and 30 and 40

42. limit 41 to yr="2006 -Current"

43. remove duplicates from 42

**MEDLINE In-Process**

1. MDD.ti,ab.

2. depress*.ti,ab.

3. 1 or 2

4. screening.ti.

5. child.mp.

6. pediatric*.mp.

7. paediatric*.mp.

8. (boy or boys).mp.

9. girl*.mp.

10. (kid or kids).mp.

11. school?age*.mp.

12. juvenil*.mp.

13. under?age*.mp.

14. teen*.mp.

15. pubescen*.mp.

16. adolescen*.mp.

17. (youth or youths).mp.

18. child*.jw.

19. pediatric*.jw.

20. paediatric*.jw.

21. adolescen*.jw.

22. or/5-21

23. random*.mp.

24. placebo*.mp.

25. trial*.mp.

26. or/23-25

27. 3 and 4 and 22 and 26

**EMBASE**

1. Depressive Disorder/

2. Depressive Disorder, Major/

3. depress*.ti.

4. or/1-3

5. Mass Screening/

6. screening.ti.

7. 5 or 6

8. Child/

9. child.mp.

10. Minors/

11. Pediatrics/

12. pediatric*.mp.

13. paediatric*.mp.

14. (boy or boys).mp.

15. girl*.mp.

16. (kid or kids).mp.

17. school?age*.mp.

18. juvenil*.mp.

19. under?age*.mp.

20. Adolescent/

21. teen*.mp.

22. pubescen*.mp.

23. adolescen*.mp.

24. (youth or youths).mp.

25. child*.jx.

26. pediatric*.jx.

27. paediatric*.jx.

28. adolescen*.jx.

29. or/8-28

30. random*.tw.

31. clinical trial*.mp.

32. exp Health Care Quality/

33. exp Treatment Outcome/

34. double blind*.mp.

35. placebo*.tw.

36. blind*.tw.

37. or/30-36

38. 4 and 7 and 29 and 37

39. limit 38 to yr="2006 -Current"

40. remove duplicates from 39

**PsycINFO**

1. "Depression (Emotion)"/

2. Major Depression/

3. depress*.ti.

4. or/1-3

5. Screening/

6. Screening Tests/

7. Psychodiagnosis/

8. Health Screening/

9. screening.ti.

10. or/5-9

11. Child Psychology/

12. child.mp.

13. Child Psychiatry/

14. Pediatrics/

15. pediatric*.mp.

16. paediatric*.mp.

17. (boy or boys).mp.

18. girl*.mp.

19. (kid or kids).mp.

20. school?age*.mp.

21. juvenil*.mp.

22. under?age*.mp.

23. teen*.mp.

24. pubescen*.mp.

25. adolescen*.mp.

26. (youth or youths).mp.

27. child*.jx.

28. pediatric*.jx.

29. paediatric*.jx.

30. adolescen*.jx.

31. or/11-30

32. control*.tw.

33. random*.tw.

34. exp Treatment/

35. or/32-34

36. 4 and 10 and 31 and 35

37. limit 36 to yr="2006 -Current"

38. remove duplicates from 37

**LILACS**

(ti:Depress$ OR mh:depression OR mh:”depressive disorder” OR mh:”depressive disorder, major”) AND (screening) AND (child$ OR teen$ OR adolescen$ OR youth$ OR pediatric$ OR paediatric$) AND (da:2006$ OR da:2007$ or da:2008$ OR da:2009$ or da:2010$ OR da:2011$ OR da:2012$)

Type of study: Controlled Clinical Trial

**CENTRAL**

#1 [(depression or depressive or MDD):ti,ab,kw in Trials](http://onlinelibrary.wiley.com/o/cochrane/searchHistory?mode=runquery&qnum=1) 26926
#2 [(screening):ti,ab,kw in Trials](http://onlinelibrary.wiley.com/o/cochrane/searchHistory?mode=runquery&qnum=2) 12177
#3 [(child* or teen* or adolescen* or youth* or boy* or girl* or juvenile* or pediatric* or paediatric*):ti,ab,kw in Trials](http://onlinelibrary.wiley.com/o/cochrane/searchHistory?mode=runquery&qnum=3) 113320
#4 [(#1 AND #2 AND #3), from 2006 to 2012](http://onlinelibrary.wiley.com/o/cochrane/searchHistory?mode=runquery&qnum=4) 121 (limit to CENTRAL = 108)

**Clinicaltrials.gov**

Advanced Search screen
Search terms: screening
Condition: Depression

Limited to
Age Group: Child
First Received: 1/1/2006 to 12/31/2012

**International Clinical Trials Registry Platform**

Advanced Search

screening (in title)
Depression OR Major Depressive Disorder (in Condition)
Search for clinical trials in children
Recruitment status is ALL
Date of registration is between: 01/01/2006 and 31/12/2012

**Search Strategies for Key Question #4**

**MEDLINE**

1. Depression/

2. Depressive Disorder/

3. Depressive Disorder, Major/

4. depress*.ti.

5. or/1-4

6. Mass Screening/

7. screening.ti.

8. Antidepressive Agents/

9. Antidepressive Agents, Second-Generation/

10. Serotonin Uptake Inhibitors/

11. Fluoxetine/

12. (fluoxetine or prozac or rapiflux or sarafem or selfemra or fontex or zacton or lovan).ti,ab.

13. Sertraline/

14. (sertraline or zoloft or lustral).ti,ab.

15. Paroxetine/

16. (paroxetine or paxil or pexevra or aropax or seroxat or sereupin).ti,ab.

17. Citalopram/

18. (citalopram or celexa or cipramil).ti,ab.

19. Escitalopram/

20. (escitalopram or lexapro or cipralex or seroplex or lexamil or lexam or entact).ti,ab.

21. Fluvoxamine/

22. (fluvoxamine or luvox).ti,ab.

23. (venlafaxine or effexor or efexor).ti,ab.

24. (duloxetine or cymbalta or ariclaim or xeristar or yentreve or duzela).ti,ab.

25. (desvenlafaxine or pristiq).ti,ab.

26. Bupropion/

27. (bupropion or wellbutrin or aplenzin or zyban or voxra or budeprion).ti,ab.

28. (mirtazapine or remeron or avanza zispin).ti,ab.

29. Trazodone/

30. (trazodone or desyrel or oleptro or beneficat or deprax or desirel or molipaxin or thombran or trazorel or trialodine or trittico or mesyrel).ti,ab.

31. Combined Modality Therapy/

32. SSRI.ti,ab.

33. SSRIs.ti,ab.

34. SNRI.ti,ab.

35. SNRIs.ti,ab.

36. serotonin uptake inhibitor*.ti,ab.

37. serotonin reuptake inhibitor*.ti,ab.

38. norepinephrine reuptake inhibitor*.ti,ab.

39. antidepress*.ti,ab.

40. anti-depress*.ti,ab.

41. pharmacologic*.ti.

42. pharmacotherap*.ti.

43. exp Psychotherapy/

44. cognitive behavio?ral.ti,ab.

45. CBT.ti,ab.

46. MBCT.ti,ab.

47. mindfulness.ti,ab.

48. family support.ti,ab.

49. family therapy.ti,ab.

50. parent* education.ti,ab.

51. Parents/ed

52. Counseling/

53. Directive Counseling/

54. counsel.ti,ab.

55. group therapy.ti,ab.

56. psychodynamic therapy.ti,ab.

57. future directed therapy.ti,ab.

58. (cognitive adj (therap* or treatment* or intervention*)).ti,ab.

59. (behavio* adj (therap* or treatment* or intervention*)).ti,ab.

60. psychotherap*.ti,ab.

61. interpersonal therap*.ti,ab.

62. interpersonal intervention*.ti,ab.

63. IPT-AST.ti,ab.

64. IPT-A.ti,ab.

65. (acceptance adj commitment therapy).ti,ab.

66. psychoeducation.ti,ab.

67. psycho education.ti,ab.

68. dynamic therapy.ti,ab.

69. (brief adj2 therap*).ti.

70. Therapy, Computer-Assisted/

71. ((internet or online or on-line or web) adj2 (therap* or treatment* or intervention*)).ti,ab.

72. Self-Care/

73. Self-Help Groups/

74. self-help.ti,ab.

75. wait* list control.ti,ab.

76. treatment as usual.ti,ab.

77. Problem Solving/

78. problem solving.ti,ab.

79. behavio* activation therapy.ti,ab.

80. exp Exercise/

81. Exercise Therapy/

82. "Physical Education and Training"/

83. Exercise Movement/

84. exercis*.ti,ab.

85. physical activit*.ti,ab.

86. or/6-85

87. Child/

88. child.mp.

89. Minors/

90. Pediatrics/

91. pediatric*.mp.

92. paediatric*.mp.

93. (boy or boys).mp.

94. girl*.mp.

95. (kid or kids).mp.

96. school?age*.mp.

97. juvenil*.mp.

98. under?age*.mp.

99. Adolescent/

100. teen*.mp.

101. pubescen*.mp.

102. adolescen*.mp.

103. (youth or youths).mp.

104. child*.jw.

105. pediatric*.jw.

106. paediatric*.jw.

107. adolescen*.jw.

108. or/87-107

109. (adverse effects or chemically induced or complications or drug effects or mortality or poisoning or toxicity).fs.

110. adverse effect*.ti,ab.

111. adverse event*.ti,ab.

112. adverse reaction*.ti,ab.

113. adverse outcome*.ti,ab.

114. Adverse drug reaction reporting systems/

115. ADRS.ti,ab.

116. Drug toxicity/

117. Drug hypersensitivity/

118. Death/

119. death*.ti,ab.

120. fatal.ti,ab.

121. Suicide/

122. Suicide, attempted/

123. suicide.ti,ab.

124. suicidal*.ti,ab.

125. mania.ti,ab.

126. manic episode*.ti,ab.

127. Overdos*.ti,ab,sh.

128. self damag*.ti,ab.

129. self injur*.ti,ab.

130. self harm.ti,ab.

131. Self injurious behavior/

132. self inflict*.ti,ab.

133. (safe or safety).ti,ab.

134. exp Diagnostic Errors/

135. misdiagnosis.ti,ab.

136. or/109-135

137. Antidepressive agents, second-generation/ae, po, to

138. Serotonin uptake inhibitors/ae, po, to

139. Fluoxetine/ae, po, to

140. Fluvoxamine/ae, po, to

141. Paroxetine/ae, po, to

142. Sertraline/ae, po, to

143. Citalopram/ae, po, to

144. Escitalopram/ae, po, to

145. Bupropion/ae, po, to

146. Trazodone/ae, po, to

147. or/137-146

148. 5 and 86 and 136

149. 147 or 148

150. 108 and 149

151. limit 150 to yr="2006 -Current"

152. remove duplicates from 151

**MEDLINE In-Process**

1. MDD.ti,ab.

2. depress*.ti,ab.

3. 1 or 2

4. screening.ti.

5. (fluoxetine or prozac or rapiflux or sarafem or selfemra or fontex or zacton or lovan).ti,ab.

6. (sertraline or zoloft or lustral).ti,ab.

7. (paroxetine or paxil or pexevra or aropax or seroxat or sereupin).ti,ab.

8. (citalopram or celexa or cipramil).ti,ab.

9. (escitalopram or lexapro or cipralex or seroplex or lexamil or lexam or entact).ti,ab.

10. (fluvoxamine or luvox).ti,ab.

11. (venlafaxine or effexor or efexor).ti,ab.

12. (duloxetine or cymbalta or ariclaim or xeristar or yentreve or duzela).ti,ab.

13. (desvenlafaxine or pristiq).ti,ab.

14. (bupropion or wellbutrin or aplenzin or zyban or voxra or budeprion).ti,ab.

15. (mirtazapine or remeron or avanza zispin).ti,ab.

16. (trazodone or desyrel or oleptro or beneficat or deprax or desirel or molipaxin or thombran or trazorel or trialodine or trittico or mesyrel).ti,ab.

17. ((combined or multimodal) adj (treatment* or intervention* or therap*)).ti,ab.

18. SSRI.ti,ab.

19. SSRIs.ti,ab.

20. SNRI.ti,ab.

21. SNRIs.ti,ab.

22. serotonin uptake inhibitor*.ti,ab.

23. serotonin reuptake inhibitor*.ti,ab.

24. norepinephrine reuptake inhibitor*.ti,ab.

25. antidepress*.ti,ab.

26. anti-depress*.ti,ab.

27. pharmacologic*.ti.

28. pharmacotherap*.ti.

29. cognitive behavio?ral.ti,ab.

30. CBT.ti,ab.

31. MBCT.ti,ab.

32. mindfulness.ti,ab.

33. family support.ti,ab.

34. family therapy.ti,ab.

35. parent* education.ti,ab.

36. counsel.ti,ab.

37. group therapy.ti,ab.

38. psychodynamic therapy.ti,ab.

39. future directed therapy.ti,ab.

40. (cognitive adj (therap* or treatment* or intervention*)).ti,ab.

41. (behavio* adj (therap* or treatment* or intervention*)).ti,ab.

42. psychotherap*.ti,ab.

43. interpersonal therap*.ti,ab.

44. interpersonal intervention*.ti,ab.

45. IPT-AST.ti,ab.

46. IPT-A.ti,ab.

47. (acceptance adj commitment therapy).ti,ab.

48. psychoeducation.ti,ab.

49. psycho education.ti,ab.

50. dynamic therapy.ti,ab.

51. (brief adj2 therap*).ti.

52. computer assisted therap*.ti,ab.

53. ((internet or online or on-line or web) adj2 (therap* or treatment* or intervention*)).ti,ab.

54. self-help.ti,ab.

55. wait* list control.ti,ab.

56. treatment as usual.ti,ab.

57. problem solving.ti,ab.

58. behavio* activation therapy.ti,ab.

59. exercis*.ti,ab.

60. physical activit*.ti,ab.

61. or/4-60

62. child.mp.

63. pediatric*.mp.

64. paediatric*.mp.

65. (boy or boys).mp.

66. girl*.mp.

67. (kid or kids).mp.

68. school?age*.mp.

69. juvenil*.mp.

70. under?age*.mp.

71. teen*.mp.

72. pubescen*.mp.

73. adolescen*.mp.

74. (youth or youths).mp.

75. child*.jw.

76. pediatric*.jw.

77. paediatric*.jw.

78. adolescen*.jw.

79. or/62-78

80. poisoning.ti,ab.

81. adverse effect*.ti,ab.

82. adverse event*.ti,ab.

83. adverse reaction*.ti,ab.

84. adverse outcome*.ti,ab.

85. adverse drug reaction*.ti,ab.

86. ADRS.ti,ab.

87. toxicity.ti,ab.

88. hypersensitivity.ti,ab.

89. death*.ti,ab.

90. fatal.ti,ab.

91. suicide.ti,ab.

92. suicidal*.ti,ab.

93. mania.ti,ab.

94. manic episode*.ti,ab.

95. Overdos*.ti,ab,sh.

96. self damag*.ti,ab.

97. self injur*.ti,ab.

98. self harm.ti,ab.

99. self inflict*.ti,ab.

100. (safe or safety).ti,ab.

101. diagnostic error*.ti,ab.

102. misdiagnosis.ti,ab.

103. or/80-102

104. 3 and 61 and 79 and 103

**EMBASE**

1. Depressive Disorder/

2. Depressive Disorder, Major/

3. depress*.ti.

4. or/1-3

5. Mass Screening/

6. screening.ti.

7. Antidepressive Agents/

8. Antidepressive Agents, Second-Generation/

9. Serotonin Uptake Inhibitors/

10. Noradrenaline Uptake Inhibitor/

11. Fluoxetine/

12. (fluoxetine or prozac or rapiflux or sarafem or selfemra or fontex or zacton or lovan).ti,ab.

13. Sertraline/

14. (sertraline or zoloft or lustral).ti,ab.

15. Paroxetine/

16. (paroxetine or paxil or pexevra or aropax or seroxat or sereupin).ti,ab.

17. Citalopram/

18. (citalopram or celexa or cipramil).ti,ab.

19. Escitalopram/

20. (escitalopram or lexapro or cipralex or seroplex or lexamil or lexam or entact).ti,ab.

21. Fluvoxamine/

22. (fluvoxamine or luvox).ti,ab.

23. (venlafaxine or effexor or efexor).ti,ab.

24. (duloxetine or cymbalta or ariclaim or xeristar or yentreve or duzela).ti,ab.

25. (desvenlafaxine or pristiq).ti,ab.

26. Bupropion/

27. (bupropion or wellbutrin or aplenzin or zyban or voxra or budeprion).ti,ab.

28. (mirtazapine or remeron or avanza zispin).ti,ab.

29. Trazodone/

30. (trazodone or desyrel or oleptro or beneficat or deprax or desirel or molipaxin or thombran or trazorel or trialodine or trittico or mesyrel).ti,ab.

31. Combined Modality Therapy/

32. SSRI.ti,ab.

33. SSRIs.ti,ab.

34. SNRI.ti,ab.

35. SNRIs.ti,ab.

36. serotonin uptake inhibitor*.ti,ab.

37. serotonin reuptake inhibitor*.ti,ab.

38. norepinephrine reuptake inhibitor*.ti,ab.

39. antidepress*.ti,ab.

40. anti-depress*.ti,ab.

41. pharmacologic*.ti.

42. pharmacotherap*.ti.

43. exp Psychotherapy/

44. cognitive behavio?ral.ti,ab.

45. CBT.ti,ab.

46. MBCT.ti,ab.

47. mindfulness.ti,ab.

48. family support.ti,ab.

49. family therapy.ti,ab.

50. parent* education.ti,ab.

51. Parents/ed

52. Counseling/

53. Directive Counseling/

54. counsel.ti,ab.

55. group therapy.ti,ab.

56. psychodynamic therapy.ti,ab.

57. future directed therapy.ti,ab.

58. (cognitive adj (therap* or treatment* or intervention*)).ti,ab.

59. (behavio* adj (therap* or treatment* or intervention*)).ti,ab.

60. psychotherap*.ti,ab.

61. interpersonal therap*.ti,ab.

62. interpersonal intervention*.ti,ab.

63. IPT-AST.ti,ab.

64. IPT-A.ti,ab.

65. (acceptance adj commitment therapy).ti,ab.

66. psychoeducation.ti,ab.

67. psycho education.ti,ab.

68. dynamic therapy.ti,ab.

69. (brief adj2 therap*).ti.

70. Therapy, Computer-Assisted/

71. ((internet or online or on-line or web) adj2 (therap* or treatment* or intervention*)).ti,ab.

72. Self-Care/

73. Self-Help Groups/

74. self-help.ti,ab.

75. wait* list control.ti,ab.

76. treatment as usual.ti,ab.

77. Problem Solving/

78. problem solving.ti,ab.

79. behavio* activation therapy.ti,ab.

80. exp Exercise/

81. Exercise Therapy/

82. "Physical Education and Training"/

83. Exercise Movement/

84. exercis*.ti,ab.

85. physical activit*.ti,ab.

86. or/5-85

87. Child/

88. child.mp.

89. Minors/

90. Pediatrics/

91. pediatric*.mp.

92. paediatric*.mp.

93. (boy or boys).mp.

94. girl*.mp.

95. (kid or kids).mp.

96. school?age*.mp.

97. juvenil*.mp.

98. under?age*.mp.

99. Adolescent/

100. teen*.mp.

101. pubescen*.mp.

102. adolescen*.mp.

103. (youth or youths).mp.

104. child*.jx.

105. pediatric*.jx.

106. paediatric*.jx.

107. adolescen*.jx.

108. or/87-107

109. (adverse drug reaction or drug toxicity).fs.

110. adverse effect*.ti,ab.

111. adverse event*.ti,ab.

112. adverse reaction*.ti,ab.

113. adverse outcome*.ti,ab.

114. Adverse drug reaction reporting systems/

115. ADRS.ti,ab.

116. Drugs/ae, to

117. Drug intoxication/

118. Drug hypersensitivity/

119. Death/

120. death*.ti,ab.

121. fatal.ti,ab.

122. Suicide/

123. Suicide, attempted/

124. suicide.ti,ab.

125. suicidal*.ti,ab.

126. mania.ti,ab.

127. manic episode*.ti,ab.

128. Overdos*.ti,ab,sh.

129. self damag*.ti,ab.

130. self injur*.ti,ab.

131. self harm.ti,ab.

132. Self injurious behavior/

133. self inflict*.ti,ab.

134. (safe or safety).ti,ab.

135. exp Diagnostic Errors/

136. misdiagnosis.ti,ab.

137. or/109-136

138. Antidepressive agents, second-generation/ae, to

139. Serotonin uptake inhibitors/ae, to

140. Fluoxetine/ae, to

141. Fluvoxamine/ae, to

142. Paroxetine/ae, to

143. Sertraline/ae, to

144. Citalopram/ae, to

145. Escitalopram/ae, to

146. Bupropion/ae, to

147. Trazodone/ae, to

148. or/138-147

149. 4 and 86 and 137

150. 148 or 149

151. 108 and 150

152. limit 151 to yr="2006 -Current"

153. remove duplicates from 152

**PsycINFO**

1. "Depression (Emotion)"/

2. Major Depression/

3. depress*.ti.

4. or/1-3

5. Screening/

6. Screening Tests/

7. Psychodiagnosis/

8. Health Screening/

9. screening.ti.

10. Antidepressant Drugs/

11. Serotonin Reuptake Inhibitors/

12. Serotonin Norepinephrine Reuptake Inhibitors/

13. Fluoxetine/

14. (fluoxetine or prozac or rapiflux or sarafem or selfemra or fontex or zacton or lovan).ti,ab.

15. Sertraline/

16. (sertraline or zoloft or lustral).ti,ab.

17. Paroxetine/

18. (paroxetine or paxil or pexevra or aropax or seroxat or sereupin).ti,ab.

19. Citalopram/

20. (citalopram or celexa or cipramil).ti,ab.

21. Escitalopram/

22. (escitalopram or lexapro or cipralex or seroplex or lexamil or lexam or entact).ti,ab.

23. Fluvoxamine/

24. (fluvoxamine or luvox).ti,ab.

25. Venlafaxine/

26. (venlafaxine or effexor or efexor).ti,ab.

27. (duloxetine or cymbalta or ariclaim or xeristar or yentreve or duzela).ti,ab.

28. (desvenlafaxine or pristiq).ti,ab.

29. Bupropion/

30. (bupropion or wellbutrin or aplenzin or zyban or voxra or budeprion).ti,ab.

31. (mirtazapine or remeron or avanza zispin).ti,ab.

32. Trazodone/

33. (trazodone or desyrel or oleptro or beneficat or deprax or desirel or molipaxin or thombran or trazorel or trialodine or trittico or mesyrel).ti,ab.

34. Multimodal Treatment Approach/

35. SSRI.ti,ab.

36. SSRIs.ti,ab.

37. SNRI.ti,ab.

38. SNRIs.ti,ab.

39. serotonin uptake inhibitor*.ti,ab.

40. serotonin reuptake inhibitor*.ti,ab.

41. norepinephrine reuptake inhibitor*.ti,ab.

42. antidepress*.ti,ab.

43. anti-depress*.ti,ab.

44. pharmacologic*.ti.

45. pharmacotherap*.ti.

46. Psychotherapy/

47. Individual Psychotherapy/

48. cognitive behavio?ral.ti,ab.

49. CBT.ti,ab.

50. MBCT.ti,ab.

51. mindfulness.ti,ab.

52. family support.ti,ab.

53. family therapy.ti,ab.

54. parent* education.ti,ab.

55. Parent Training/

56. Counseling/

57. counsel.ti,ab.

58. group therapy.ti,ab.

59. psychodynamic therapy.ti,ab.

60. future directed therapy.ti,ab.

61. (cognitive adj (therap* or treatment* or intervention*)).ti,ab.

62. (behavio* adj (therap* or treatment* or intervention*)).ti,ab.

63. psychotherap*.ti,ab.

64. interpersonal therap*.ti,ab.

65. interpersonal intervention*.ti,ab.

66. IPT-AST.ti,ab.

67. IPT-A.ti,ab.

68. (acceptance adj commitment therapy).ti,ab.

69. psychoeducation.ti,ab.

70. psycho education.ti,ab.

71. dynamic therapy.ti,ab.

72. (brief adj2 therap*).ti.

73. Computer Assisted Therapy/

74. ((internet or online or on-line or web) adj2 (therap* or treatment* or intervention*)).ti,ab.

75. Self-Care/

76. Self-Help Techniques/

77. self-help.ti,ab.

78. wait* list control.ti,ab.

79. treatment as usual.ti,ab.

80. Problem Solving/

81. problem solving.ti,ab.

82. behavio* activation therapy.ti,ab.

83. exp Exercise/

84. exercis*.ti,ab.

85. physical activit*.ti,ab.

86. or/5-85

87. Child Psychology/

88. child.mp.

89. Child Psychiatry/

90. Pediatrics/

91. pediatric*.mp.

92. paediatric*.mp.

93. (boy or boys).mp.

94. girl*.mp.

95. (kid or kids).mp.

96. school?age*.mp.

97. juvenil*.mp.

98. under?age*.mp.

99. teen*.mp.

100. pubescen*.mp.

101. adolescen*.mp.

102. (youth or youths).mp.

103. child*.jx.

104. pediatric*.jx.

105. paediatric*.jx.

106. adolescen*.jx.

107. or/87-106

108. Treatment Effectivess Evaluation/

109. adverse effect*.ti,ab.

110. adverse event*.ti,ab.

111. adverse reaction*.ti,ab.

112. adverse outcome*.ti,ab.

113. adverse drug reaction*.ti,ab.

114. ADRS.ti,ab.

115. "Side Effects (Drug)"/

116. "Side Effects (Treatment)"/

117. Toxicity/

118. Drug Sensitivity/

119. "Death and Dying"/

120. Mortality Rate/

121. death*.ti,ab.

122. fatal.ti,ab.

123. Suicide/

124. Attempted Suicide/

125. Suicidal Ideation/

126. suicide.ti,ab.

127. suicidal*.ti,ab.

128. mania.ti,ab.

129. manic episode*.ti,ab.

130. Overdos*.ti,ab,sh.

131. self damag*.ti,ab.

132. self injur*.ti,ab.

133. self harm.ti,ab.

134. exp Self injurious behavior/

135. self inflict*.ti,ab.

136. Safety/

137. (safe or safety).ti,ab.

138. Misdiagnosis/

139. misdiagnosis.ti,ab.

140. or/108-139

141. 4 and 86 and 107 and 140

142. limit 141 to yr="2006 -Current"

143. remove duplicates from 142

**CENTRAL**

#1 [(depression or depressive or MDD):ti,ab,kw in Trials](http://onlinelibrary.wiley.com/o/cochrane/searchHistory?mode=runquery&qnum=1) 26926

#2 [(screening or treatment* or intervention* or psychotherapy or therap* or pharmacotherap* or drug* or SSRI* or SNRI* or anti-depress* or antidepress* or fluoxetine or sertraline or paroxetine or citalopram or escitalopram or fluvoxamine or venlafaxine or duloxetine or desvenlafaxine or bupropion or mirtazapine or trazodone or counsel* or exercis*):ti,ab,kw in Trials](http://onlinelibrary.wiley.com/o/cochrane/searchHistory?mode=runquery&qnum=2) 465448

#3 [(child* or teen* or adolescen* or youth* or boy* or girl* or juvenile* or pediatric* or paediatric*):ti,ab,kw in Trials](http://onlinelibrary.wiley.com/o/cochrane/searchHistory?mode=runquery&qnum=3) 113320

#4 [(adverse or side effect* or fatal* or death* or suicid* or toxicity or overdos* or mania or manic episode or self-harm or self-injur* or self damag* or self inflict* or safety or misdiagnos* ):ti,ab,kw in Trials](http://onlinelibrary.wiley.com/o/cochrane/searchHistory?mode=runquery&qnum=4) 174864

#5 [(#1 AND #2 AND #3 AND #4), from 2006 to 2012](http://onlinelibrary.wiley.com/o/cochrane/searchHistory?mode=runquery&qnum=5)

**LILACS**

(ti:Depress$ OR mh:depression OR mh:"depressive disorder" OR mh:"depressive disorder, major") AND (screening OR treatment$ OR intervention$ OR psychotherapy OR therap$ OR pharmacotherapy$ OR drug$ OR SSRI$ OR SNRI$ OR anti-depress$ OR antidepress$ OR fluoxetine OR sertraline OR paroxetine OR citalopram OR escitalopram OR fluvoxamine OR venlafaxine OR duloxetine OR desvenlafaxine OR bupropion OR mirtazapine OR trazodone OR counsel$ OR exercis$) AND (adverse OR side effect$ OR fatal$ OR death$ OR suicid$ OR toxicity OR overdos$ OR mania OR manic episode OR self-harm OR self-injur$ OR self damag$ OR self inflict$ OR safety OR misdiagnos$) AND (child$ OR teen$ OR adolescen$ OR youth$ OR pediatric$ OR paediatric$) AND (da:2006$ OR da:2007$ OR da:2008$ OR da:2009$ OR da:2010$ OR da:2011$ OR da:2012$)

**Clinicaltrials.gov**

Advanced Search screen
Search terms: harm OR harms OR adverse effects OR side effects OR suicide OR fatal OR death OR toxicity OR overdos* OR manic OR mania OR self injury OR safety OR misdiagnosis
Condition: Depression

Limited to
Age Group: Child
First Received: 1/1/2006 to 12/31/2012

**International Clinical Trials Registry Platform**

Advanced Search

harm* OR injur* OR adverse OR side effect* OR toxicity OR safety OR suicid* OR damag* OR misdiagnos* OR death* OR fatal* OR overdos* (in title)
Depression OR Major Depressive Disorder (in Condition)
Search for clinical trials in children
Recruitment status is ALL
Date of registration is between: 01/01/2006 and 31/12/2012
