# Supplementary material for: Depression screening and mental health outcomes in children and adolescents: a systematic review protocol
Source: Syst Rev. 2012 Nov 24;1:58. doi: 10.1186/2046-4053-1-58 (PMC3563607; doi:10.1186/2046-4053-1-58)
Supplement: Additional file 3 — Coding manual. [file 2046-4053-1-58-S3.docx]

**APPENDIX 3: Coding Manual**

**Title/Abstract Review**

**KQ #1 - Accuracy of screening instruments**

**No: no original data or case study.** It is clear from the title/abstract that the article is not an original report of a study, but for example a letter, editorial, systematic review or meta-analysis, or it is a case series or case report study. Studies reporting only on animal, cellular, or genetic data are also excluded. Conference or symposium abstracts are also excluded.

**No: no children or adolescents.** The study sample does not consist of children and adolescents,

(aged 6-18). Eligible settings include general medicine clinics (e.g., pediatric or primary care and family medicine settings), schools, and community settings. Studies including medically-ill children are eligible. Studies conducted in high-risk populations where many or most children and adolescents may have a psychiatric disorder, such as in psychiatric or youth-protection settings, will be excluded. Studies of college and university populations will also be excluded. Studies with mixed population samples are included if data for children or adolescents aged 6-18 were reported separately.

**No: sample selection is based on the presence of distress or depression.** When the

study sample is a pre-selection of distressed or depressed children or adolescents (e.g., based on clinician’s judgment or screening instrument cut-off), the study is excluded. Studies in which only part of the sample is selected based on distress or depression may be included.

**No: no major depression.** Only studies that assess children or adolescents for a DSM diagnosis of MDD or ICD diagnosis of depressive episode will be included, as established using a validated structured or semi-structured clinical interview. Minor depression and dysthymia are excluded. Studies that include children or adolescents from broader diagnostic categories, such as other depressive or anxiety disorders, are included only if they report data for children or adolescents with MDD/a depressive episode separately. It is unlikely that studies can be excluded based on differential diagnosis (e.g., major versus major + minor depression) at the title/abstract level. However, it will be possible to exclude studies that clearly only used a self-report questionnaire and did not include a diagnostic interview for depression. See the instruments list for examples of eligible diagnostic interviews and depression screeners/questionnaires.

**No: no comparison of depression screening instrument with validated structured or semi-structured interview for major depression.** To be included, the study must report data on the comparison of a self-report depression screening instrument and a validated structured or semi-structured diagnostic interview for MDD/depressive episode. Any self-report questionnaire (e.g., self-rating scale, one-item screener, visual analogue scale, etc.) can be used as a depression screening instrument. At the title/abstract review, a study should only be excluded if it is clear that it did not administer both a diagnostic interview for depression and a screener/questionnaire. See the instruments list for examples of diagnostic interviews and depression screeners/questionnaires. If the abstract suggests the study may have administered both of these, select the study for full-text review, even if the abstract does not report diagnostic accuracy data (e.g., sensitivity, specificity).

**Yes:** Study eligible to be included in full-text review.

**Notes:**

- Highlight duplicate studies
- Screening tools not listed in the instruments file may still be accepted
- Highlight systematic reviews and meta-analysis on the accuracy of depression screening instruments in children and adolescents (excluded as “no original data). These will be added to the appendix list of reviews and checked for eligible studies.
- Highlight conference and symposium abstracts (excluded as “no original data”) if they report data from a potentially eligible study. The full published or unpublished study will be sought out.

**KQ #2 – Treatment**

**No: no original data or case study.** It is clear from the title/abstract that the article is not an original report of a study, but for example a letter, editorial, systematic review or meta-analysis, or it is a case series or case report study. Studies reporting only on animal, cellular, or genetic data are also excluded. Conference or symposium abstracts are also excluded.

**No: no children or adolescents.** The study sample does not consist of children and adolescents,

(aged 6-18). Eligible settings include general medicine clinics (e.g., pediatric or primary care and family medicine settings), schools, and community settings. Studies including medically-ill children are eligible. Studies of college and university populations will also be excluded. Studies with mixed population samples are included if data for children or adolescents aged 6-18 were reported separately.

**No: no RCT of treatment of depression or depressive symptoms.** It is clear from the title/abstract that the study is not an RCT of a depression treatment (pharmacological, psychotherapeutic, or other intervention) designed to reduce depressive symptoms. Eligible interventions include SSRIs (i.e., fluoxetine, sertraline, paroxetine, citalopram, escitalopram, and fluvoxamine), SNRIs (i.e., venlafaxine, duloxetine, desvenlafaxine), other non-SSRIs prescribed for children or adolescents with depression (buproprion, mirtazapine, trazadone), psychotherapy, educational interventions, and exercise. A depression treatment group must be compared to a depression control group (e.g., usual care, waiting list, attention control, placebo). Studies with enhanced usual care (such as providing information to patients and/or physicians) can be included. Studies that are head-to-head comparison studies of two active treatments (e.g., drug versus drug, drug versus psychotherapy) are not included. When the primary outcome is not major depression but, for example, a proxy for depression (e.g. SF-36 Mental Component Summary score) the study may be included. Studies with primary outcomes that reflect an intention to treat a different problem (e.g. pain, anxiety, fatigue) should be excluded.

**No: no major depression eligibility criterion.** Only studies on the treatment of major depression will be included, as established using a validated structured or semi-structured clinical interview, based on DSM or ICD criteria. Major depression must be an eligibility criterion for the study. Studies that include only children or adolescents with minor depression, dysthymia or a score above a cut-off on a self-report questionnaire are excluded. When studies include children or adolescents from broader diagnostic categories, such as any depressive disorder, they are included only if data for children or adolescents with major depression are reported separately. At the title/abstract stage, it will be difficult to exclude based on differential diagnoses (e.g. major versus major + minor depression), but it will be possible to exclude in some cases because the eligibility criterion is clearly a score on a self-report measure of depressive symptoms. See instruments list for examples of diagnostic interviews and depression screeners/questionnaires.

**Yes:** study eligible to be included in full text review.

**Notes:**

- Highlight duplicate studies
- Highlight systematic reviews and meta-analysis on the accuracy of depression treatment in children and adolescents (excluded as “no original data). These will be added to the appendix list of reviews and checked for eligible studies.
- Highlight conference and symposium abstracts (excluded as “no original data”) if they report data from a potentially eligible study. The full published or unpublished study will be sought out.
- Highlight study protocols for potentially eligible treatment studies. The full published or unpublished study will be sought out.

**KQ #3 – Screening Outcomes**

**No: no original data or case study.** It is clear from the title/abstract that the article is not an original report of a study, but for example a letter, editorial, systematic review or meta-analysis, or it is a case series or case report study. Studies reporting only on animal, cellular, or genetic data are also excluded. Conference or symposium abstracts are also excluded.

**No: no children or adolescents.** The study sample does not consist of children and adolescents,

(aged 6-18). Eligible settings include general medicine clinics (e.g., pediatric or primary care and family medicine settings), schools, and community settings. Studies including medically-ill children are eligible. Studies conducted in high-risk populations where many or most children and adolescents may have a psychiatric disorder, such as in psychiatric or youth-protection settings, will be excluded. Studies of college and university populations will also be excluded. Studies with mixed population samples are included if data for children or adolescents aged 6-18 were reported separately.

**No: no RCT or CT of screening for depression.** The study needs to be a randomized controlled trial in which the intervention group children or adolescents are screened for depression and the control group is not screened. Both groups must have access to the same or similar depression treatment options, either through screening or physician referral, to avoid confounding of screening and treatment. Depression, as verified by a validated semi-structured diagnostic interview, or depressive symptoms based on self-report measures must be an outcome of the study.

**Yes**. Study eligible to be included in full text review.

**Notes:**

- Highlight duplicate studies
- Screening tools not listed in the instruments file may still be accepted
- Highlight conference and symposium abstracts (excluded as “no original data”) if they report data from a potentially eligible study. The full published or unpublished study will be sought out.
- Highlight study protocols for potentially eligible screening studies. The full published or unpublished study will be sought out.
- Highlight excluded trials that evaluate a depression screening program in children or adolescents, but that do not meet our inclusion criteria (e.g. pre-post studies where a cohort is examined prior to implementing screening and another cohort of children or adolescents who received a screening program were examined). These will be added to a list of excluded studies for KQ#3.

**KQ #4 – Harms**

To be determined**Full-Text Review**

**KQ #1 - Accuracy of screening instruments**

**No: no original data or case study.** The article is not an original report of a study, but for example a letter, editorial, study protocol, systematic review or meta-analysis, or it is a case series or case report study. Conference or symposium abstracts are also excluded.

**No: no children or adolescents.** The study sample does not consist of children and adolescents,

(aged 6-18). Eligible settings include general medicine clinics (e.g., pediatric or primary care and family medicine settings), schools, and community settings. Studies including medically-ill children are eligible. Studies conducted in high-risk populations where many or most children and adolescents may have a psychiatric disorder, such as in psychiatric or youth-protection settings, will be excluded. Studies of college and university populations will also be excluded. Studies with mixed population samples are included if data for children or adolescents aged 6-18 were reported separately.

**No: sample selection is based on the presence of distress or depression.** When the

study sample is a pre-selection of distressed or depressed children or adolescents (e.g., based on clinician’s judgment or screening instrument cut-off), the study is excluded. Studies in which only part of the sample is selected based on distress or depression may be included.

**No: no major depression.** Only studies that assess children or adolescents for a DSM diagnosis of MDD or ICD diagnosis of depressive episode will be included, as established using a validated structured or semi-structured clinical interview. If it is reported that MDD was assessed using a semi-structured or fully-structured interview, but the name of the interview is not reported, the study is excluded. Minor depression and dysthymia are excluded. Studies that include children or adolescents from broader diagnostic categories, such as other depressive or anxiety disorders, are included only if they report data for children or adolescents with MDD separately.

*Examples of inclusion / exclusion of different depression diagnoses*:

**DSM-IV-TR:** Include: Major Depression. Exclude: Dysthymic Disorder. Exclude: Minor Depression (at least two depressive symptoms are present for two weeks).

**ICD-10:** Include: mild, moderate, severe, recurrent depressive episodes. Exclude: recurrent brief depressive disorder (requires a depressive episode with symptomatic criteria, but lasting less than 2 weeks and requires that the episodes occur at least once per month for 12 consecutive months).

**RESEARCH DIAGNOSTIC CRITERIA (RDC):** Include: Major Depressive Disorder.

**DSM-III:** Include: Major depression. Exclude: Dysthymic Disorder. Exclude: atypical affective disorders.

**No: no comparison of depression screening instrument with validated structured or semi-structured interview for major depression.** To be included, the study must

report data on the comparison of a depression screening instrument and a validated structured or semi-structured diagnostic interview for major depressive disorder. Any self-report questionnaire (e.g., self-rating scale, one-item screener, visual analogue scale, etc) can be used as a depression screening instrument. Studies in which only parent or teacher-completed measures, and no child or adolescent self-report measures, are compared to a diagnosis of MDD/depressive episode will be excluded. Sufficient data must be reported to complete a basic 2x2 table (see below), and we will not contact study authors for additional data. The screening instrument and diagnostic interview must be administered within two weeks of one another.

|  | Screening score + | Screening score - | Total |
| --- | --- | --- | --- |
| Depression + |  |  | Total depressed |
| Depression - |  |  | Total not depressed |
| Total | Total screened positive | Total screened negative | Total sample |

**Yes:** Study eligible to be included in full-text review.

**Notes:**

- Screening tools not listed in the instruments file may still be accepted
- Highlight conference and symposium abstracts (excluded as “no original data”) if they report data from a potentially eligible study. The full published or unpublished study will be sought out.

**KQ #2 – Treatment**

**No: no original data or case study.** The article is not an original report of a study, but for example a letter, editorial, study protocol, systematic review or meta-analysis, or it is a case series or case report study. Conference or symposium abstracts are also excluded..

**No: no children or adolescents.** The study sample does not consist of children and adolescents,

(aged 6-18). Eligible settings include general medicine clinics (e.g., pediatric or primary care and family medicine settings), schools, and community settings. Studies including medically-ill children are eligible. Studies of college and university populations will also be excluded. Studies with mixed population samples are included if data for children or adolescents aged 6-18 were reported separately.

**No: no RCT of treatment of depression or depressive symptoms.** The study is not a randomized controlled trial of a depression treatment (pharmacological, psychotherapeutic, or other intervention) designed to reduce depressive symptoms, as opposed to medical treatments designed to treat physical symptoms. Eligible interventions include SSRIs (i.e., fluoxetine, sertraline, paroxetine, citalopram, escitalopram, and fluvoxamine), SNRIs (i.e., venlafaxine, duloxetine, desvenlafaxine), other non-SSRIs prescribed for children or adolescents with depression (buproprion, mirtazapine, trazadone), psychotherapy, educational interventions, and exercise. A depression treatment group must be compared to a depression control group (e.g., usual care, waiting list, attention control, placebo). Studies that are head-to-head comparison studies of two active treatments (e.g., drug versus drug, drug versus psychotherapy) are excluded. Studies with enhanced usual care (such as providing information to patients and/or physicians) can be included. Studies with control groups in which there is any active intervention, such as receiving attention from a provider even if the attention was hypothesized to be inert, are excluded. When the primary outcome is not major depression but, for example, a proxy for depression (e.g. SF-36 Mental Component Summary score) the study may be included. Studies with primary outcomes that reflect an intention to treat a different problem (e.g. pain, anxiety, fatigue) are excluded.

**No: no major depression eligibility criterion.** Only studies on the treatment of major depression will be included, as established using a validated structured or semi-structured clinical interview, based on DSM or ICD criteria. Major depression must be an eligibility criterion for the study. Studies that include only children or adolescents with minor depression, dysthymia or a score above a cut-off on a self-report questionnaire are excluded. When studies include children or adolescents from broader diagnostic categories, such as other depressive disorders, they are included only if data for children or adolescents with major depression are reported separately.

**Yes:** study eligible to be included in full text review.

**Notes:**

- Highlight conference and symposium abstracts (excluded as “no original data”) if they report data from a potentially eligible study. The full published or unpublished study will be sought out.
- Highlight study protocols for potentially eligible treatment studies. The full published or unpublished study will be sought out.

**KQ #3 – Screening Outcomes**

**No: no original data or case study.** The article is not an original report of a study, but for example a letter, editorial, study protocol, systematic review or meta-analysis, or it is a case series or case report study. Conference or symposium abstracts are also excluded.

**No: no children or adolescents.** The study sample does not consist of children and adolescents,

(aged 6-18). Eligible settings include general medicine clinics (e.g., pediatric or primary care and family medicine settings), schools, and community settings. Studies including medically-ill children are eligible. Studies conducted in high-risk populations where many or most children and adolescents may have a psychiatric disorder, such as in psychiatric or youth-protection settings, will be excluded. Studies of college and university populations will also be excluded. Studies with mixed population samples are included if data for children or adolescents aged 6-18 were reported separately.

**No: no RCT or CT of screening for depression.** The study needs to be a randomized controlled trial in which the intervention group is screened for depression and the control group is not screened. A cutoff on a depression screening tool that would be used to identify possible cases and determine decisions regarding further assessment or treatment must be defined a priori. Studies in which questionnaire results were provided to clinicians without guidance on cut-off scores to determine positive screening status and further assessment or treatment are also excluded. Studies that administered multiple screening tools for multiple problems may be included if all of the measures have defined cutoffs for positive screens and all are screens depression. Both groups must have access to the same or similar depression treatment options, either through screening or physician referral, to avoid confounding of screening and treatment. Depression diagnoses, as verified by a validated semi-structured diagnostic interview, or depressive symptoms based on self-report measures must be an outcome of the study. Studies that include only case identification or depression treatment rates as outcomes are excluded.

**Yes**. Study eligible to be included in full text review.

**Notes:**

- Screening tools not listed in the instruments file may still be accepted
- Highlight conference and symposium abstracts (excluded as “no original data”) if they report data from a potentially eligible study. The full published or unpublished study will be sought out.
- Highlight study protocols for potentially eligible screening studies. The full published or unpublished study will be sought out.
- Highlight excluded trials that evaluate a depression screening program in children or adolescents, but that do not meet our inclusion criteria (e.g. pre-post studies where a cohort is examined prior to implementing screening and another cohort of children or adolescents who received a screening program were examined). We will include these in an excluded studies list. These will be added to a list of excluded studies for KQ#3.

**KQ #4 – Harms**

To be determined
