# Supplementary material for: Depression screening and mental health outcomes in children and adolescents: a systematic review protocol
Source: Syst Rev. 2012 Nov 24;1:58. doi: 10.1186/2046-4053-1-58 (PMC3563607; doi:10.1186/2046-4053-1-58)
Supplement: Additional file 4 — Variables included in data extraction form. [file 2046-4053-1-58-S4.docx]

**APPENDIX 4: Variables Included in Data Extraction Form**

**Key Question #1 (accuracy of screening tools)**

Authors

Year

Country

Age group

Setting

Sample characteristics notes

Key inclusion criteria

Key exclusion criteria

Number of patients

Recruitment rate of eligible patients

Mean age

Percent male

Structured interview used (MDD criterion standard)

Number (%) with major depressive disorder

Screening tool and cut-off threshold

Derivation of cut-off (e.g., literature, exploratory)

Range of cut-offs reported

Number (%) above threshold on screening tool

Interviewer blinded to screening results?

Order of administration (structured interview versus screening tool)

Number positive MDD/positive screening tool

Number positive MDD/negative screening tool

Number negative MDD/positive screening tool

Number negative MDD/negative screening tool

Sensitivity

Specificity

Positive predictive value

Negative predictive value

Notes

**Key Question #2 (effects of treatment)**

Authors

Year

Country

Published trial design preceded results?

Trial registration

Publication status (e.g., published, unpublished)

Source, if unpublished

Study funding source

Number of authors with conflicts of interest (COIs) / Total number of authors

Age group

Setting

Sample characteristics notes

Key inclusion criteria

Key exclusion criteria

Mean age

Percent male

Structured interview used (MDD criterion for trial entry)

Treatment

Treatment protocol (e.g., dose, hours per week)

Control group (e.g., UC, placebo)

Weeks of treatment

N intent to treat

N treatment

N control

For each depression outcome variable:

Outcome variable

Effect size measure (e.g., d, g, r)

Effect size (+ for treatment better)

Intent to treat?

**Key Question #3 (effects of screening)**

Author

Year

Country

Published trial design preceded results?

Trial registration

Study funding source

Number of authors with conflicts of interest (COIs) / Total number of authors

Age group

Setting

Sample characteristics notes

Key inclusion criteria

Key exclusion criteria

Mean age

Percent male

N treatment

N control

Treatment / intervention procedure

Control condition

Screening instrument and cutoff

Method to diagnose MDD

Follow-up time

Primary outcome measure

Effect size

N drop out / lost to follow-up

**Key Question #4 (harms)**

To be determined
